# P300 Event-Related Potentials as Cognitive Biomarkers in Neurological and Neuropsychiatric Disorders: A Systematic Review

**DOI:** 10.31083/RN49664

**Published:** 2026-05-26

**Authors:** Evgenia Gkintoni, Apostolos Vantarakis, Philippos Gourzis

**Affiliations:** ^1^Department of Medicine, University of Patras, 26504 Patras, Greece; ^2^Department of Psychiatry, University General Hospital of Patras, 26504 Patras, Greece

**Keywords:** P300, event-related potentials, cognitive biomarkers, electroencephalography, transcranial direct current stimulation, transcranial magnetic stimulation, neuropsychiatric disorders

## Abstract

**Background:**

Neurological and neuropsychiatric disorders constitute a major global health challenge. The P300 event-related potential, an electroencephalography-derived measure of cognitive processing, has emerged as a promising biomarker for diagnosis, treatment monitoring, and outcome prediction. This systematic review examines P300's role across neurological and neuropsychiatric disorders, evaluating how P300 latency (processing speed) and amplitude (attentional resource allocation) may reflect neurocognitive dysfunction.

**Methods:**

Following Preferred Reporting Items for Systematic Reviews and Meta-Analyses (PRISMA) 2020 guidelines, we searched PubMed, Scopus, and Web of Science for randomized controlled trials and controlled studies published January 2020–August 2025. Six research domains were examined: dementia spectrum disorders (Research Question, RQ1), acquired brain injury and disorders of consciousness (RQ2), mood and anxiety disorders (RQ3), neurodevelopmental disorders (RQ4), psychotic disorders and addiction (RQ5), and chronic neurological conditions (RQ6). Risk of bias was assessed using a modified Cochrane tool. Of 125 records identified, 52 studies met the inclusion criteria.

**Results:**

P300 emerged as a promising transdiagnostic biomarker. Prolonged latency and reduced amplitude consistently characterized clinical populations versus controls (pooled standardized mean difference [SMD] = –0.72, 95% confidence interval [CI]: –0.89 to –0.55; I^2^ = 67.3%). In dementia spectrum disorders, P300 latency distinguishes mild cognitive impairment from healthy aging. In disorders of consciousness, the emergence of the P300 waveform provided objective indices of residual cognitive capacity. In mood disorders, baseline amplitude predicted therapy outcomes. In addition, P300 correlated with craving severity. Neuromodulation (transcranial direct current stimulation [tDCS], repetitive transcranial magnetic stimulation [rTMS]) produced the most robust normalization effects across categories. P300 changes occurred within 3 days to 6 weeks of treatment, potentially enabling earlier detection of response than conventional assessments. Portable electroencephalography (EEG) systems demonstrated adequate sensitivity for clinical applications.

**Conclusions:**

P300 shows promise as a non-invasive biomarker for cognitive dysfunction across neuropsychiatric disorders. Its diagnostic utility, treatment responsiveness, and prognostic potential support clinical translation. Near-term applications include consciousness assessment, cognitive screening in dementia, and treatment monitoring. However, standardization of protocols, multi-site validation, and scalable technologies require further development. Advancing P300 research through interdisciplinary collaboration may contribute to precision psychiatry and population-level mental health strategies.

## 1. Introduction

The prevention of disease and disability is a major concern for health policy. Prevention will be most successful where there is knowledge of what causes the disorder, where it can be eliminated or reduced, and where those at risk of developing the disorder can be identified using risk indicators [1, 2, 3]. Neuropsychiatric disorders are recognized as the largest contributor to global disability and have a profound impact on individuals, families, and healthcare providers due to the large number of people affected and the high cost of providing care [4, 5]. Approximately one in every four adult and child population will develop a mental disorder at some time in their life, indicating an immediate need for effective identification, tracking, and interventions [6, 7, 8, 9].

An overarching and comprehensive understanding of how the brain-body-mind-environment interface functions is needed to inform public health policy initiatives for prevention and early intervention. Recent advancements in neuroimaging and data analysis capabilities provide researchers and clinicians with new opportunities to examine the brain’s detailed structural and functional properties [10, 11, 12, 13]. Since neuroimaging data, whether obtained through magnetic resonance imaging (MRI), functional MRI (fMRI), positron emission tomography (PET), magnetoencephalography (MEG), electroencephalography (EEG) etc., are considered to be among the most promising sources of biomarkers for major brain disorders, however, there exist several important methodological barriers to realize the full potential of neuroimaging-derived biomarkers in clinical psychiatry and neurology [14, 15, 16]. These include: the complex and heterogeneous nature of data; the need for cross-validated, multi-dataset analyses across different populations; the need for the assessment of methodologies independently of developers and manufacturers; the need for thorough evaluation of accuracy, clear data descriptions, and the minimization of reporting bias. Addressing each of these challenges will be necessary to ensure the successful translation of neuroimaging-derived biomarkers for clinical use [17, 18, 19].

Among non-invasive neuroimaging techniques, EEG is unique for its low cost and portability, as well as its ability to capture the temporal dynamics of neural activity related to cognitive and emotional processing. Within the EEG domain, Event-Related Potentials (ERP) provide a temporally precise, objective measure of cognitive processing [20, 21, 22, 23]. The P300 component, a positive deflection occurring approximately 250–500 milliseconds after stimulus onset, has become one of the most widely researched ERP measures of cognition. The P300 reflects higher-order cognitive processes, including attention allocation, contextual updating, working memory engagement, and decision-making, making it particularly suitable for assessing cognitive functioning across many clinical populations [24, 25, 26, 27].

P300 has been shown to be a useful cognitive biomarker across various neurological and psychiatric disorders. For example, in neurodegenerative disorders such as Alzheimer’s Disease (AD), vascular cognitive impairment (VCI), and mild cognitive impairment (MCI), P300 abnormalities such as prolonged latency and decreased amplitude have been found to be predictive of cognitive decline, disease progression, and treatment response. Similar associations have been found between P300 and prognosis for recovery of consciousness and functional outcomes in Acquired Brain Injury and Disorders of Consciousness. Additionally, in mood and anxiety disorders, schizophrenia, addiction, attention-deficit/hyperactivity disorder (ADHD), and other psychiatric disorders, P300 alterations have been found to correlate with symptom severity, cognitive dysfunction, and therapeutic response, supporting its potential as a transdiagnostic biomarker of cognitive impairment [28, 29, 30, 31, 32].

Significant enhancements in EEG acquisition and analysis capabilities over the last ten years have made P300-based biomarkers more clinically applicable than ever before. Developments in machine learning techniques, portable EEG devices, and multimodal fusion techniques have increased the potential for applying P300-based biomarkers in the “real world” clinical setting [33, 34, 35]. There is an increasing need to use machine learning-based feature selection methods (Recursive Feature Elimination, Elastic Net Regression, and Mutual Information Ranking) to identify meaningful neural features from large-scale EEG data in a scalable, reproducible manner. Using cross-validation and separate test sets can help improve the reliability and generalizability of EEG-based biomarkers. Combining EEG with other neuroimaging modalities (fMRI, MEG, PET) can also provide additional insight into the neurobiological basis of neurological and psychiatric conditions; however, the standardization of quantitative methodologies for combining multiple imaging modalities continues to evolve [36, 37, 38, 39].

Despite a growing body of literature on the use of the P300 as a cognitive biomarker, there is currently no comprehensive framework to synthesize the approaches and results of individual studies in a manner that supports translational research [40, 41, 42, 43, 44]. This systematic review aims to address these gaps by examining empirical evidence from randomized controlled trials published between January 2020 and August 2025 to evaluate the potential utility of the P300 ERP as a cognitive biomarker for the diagnosis, management, and treatment of various clinical conditions. In particular, the systematic review focused on methodological quality, reproducibility of results, relationships between findings and other neuroimaging modalities, and the ability to scale up applications to facilitate early identification, risk assessment, and tailored treatments across diverse patient groups [45, 46, 47, 48].

This systematic review synthesizes findings from 52 randomized and controlled EEG studies to address the following key research questions [RQs]:

[RQ1] Can P300 event-related potentials serve as cognitive biomarkers for diagnosis, progression monitoring, and treatment response in Dementia Spectrum Disorders (Alzheimer’s disease, vascular cognitive impairment/vascular dementia, mild cognitive impairment)?

This question addresses the utility of P300 for detecting early cognitive changes, tracking disease progression, and evaluating therapeutic interventions in neurodegenerative dementia populations.

[RQ2] Can P300 event-related potentials serve as cognitive biomarkers for consciousness recovery, cognitive rehabilitation outcomes, and functional prognosis in Acquired Brain Injury and Disorders of Consciousness?

This question explores the prognostic value of P300 for predicting recovery trajectories and rehabilitation outcomes in post-stroke cognitive impairment, traumatic brain injury, minimally conscious states, and prolonged disorders of consciousness.

[RQ3] Can P300 event-related potentials serve as cognitive biomarkers for symptom severity, treatment prediction, and cognitive dysfunction in Mood, Anxiety, and Stress-Related Disorders?

This question examines whether P300 markers can identify cognitive correlates of affective symptoms and predict therapeutic response in major depressive disorder, anxiety disorders, obsessive-compulsive disorder, Tourette syndrome, and eating disorders.

[RQ4] Can P300 event-related potentials serve as cognitive biomarkers for attention deficits, treatment response, and neurodevelopmental outcomes in Neurodevelopmental and Attention Disorders?

This question investigates the utility of P300 for characterizing attention deficits and monitoring intervention effects in attention-deficit/hyperactivity disorder, autism spectrum disorder, and developmental dyslexia.

[RQ5] Can P300 event-related potentials serve as cognitive biomarkers for cognitive control deficits, craving, and treatment monitoring in Psychotic Disorders and Addiction?

This question evaluates P300 as a marker of cognitive control impairments and cue reactivity in schizophrenia, alcohol use disorder, substance use disorders, internet gaming disorder, smartphone addiction, and nicotine dependence.

[RQ6] Can P300 event-related potentials serve as cognitive biomarkers for cognitive dysfunction associated with Chronic Neurological and Medical Conditions?

This question assesses P300 utility for detecting and monitoring cognitive impairment secondary to multiple sclerosis, epilepsy, vestibular disorders, hepatic encephalopathy, and chronic obstructive pulmonary disease.

In addressing these research questions, this review evaluates the methodological rigor and reproducibility of P300 findings, the integration of EEG with other neuroimaging modalities, and the translational potential of these tools in real-world public health and clinical settings. The ultimate goal is to provide an evidence-based framework for implementing P300-based cognitive biomarkers in practical public health strategies for neuropsychiatric care, supporting scalable applications for early detection, risk stratification, and personalized intervention across diverse populations.

## 2. Materials and Methods

This systematic review aims to develop a comprehensive overview of the current literature regarding the use of P300 ERPs as cognitive biomarkers in patients with neurological and psychiatric disorders. This review will integrate information from three different disciplines including Clinical Neuroscience; Neuropsychiatry; and Cognitive Electrophysiology to identify P300 derived biomarkers that are related to cognitive dysfunction in multiple clinical populations, to evaluate the potential for these biomarkers as both diagnostic and predictive tools for disease progression, and to evaluate the translational potential of the P300 for clinical application including treatment monitoring and development of tailored interventions for individual patients.

### 2.1 Analytical Search Process

This review followed Preferred Reporting Items for Systematic Reviews and Meta-Analyses (PRISMA) guidelines to ensure methodological rigor and transparency (**Supplementary Materials-PRISMA 2020 Checklist**) [49, 50]. A review protocol detailing the inclusion/exclusion criteria, search strategy, and data extraction procedures was developed prior to conducting the systematic search. This systematic review was not prospectively registered in the International Prospective Register of Systematic Reviews (PROSPERO) or other systematic review registries. The review protocol was developed a priori, specifying inclusion and exclusion criteria, search strategy, data extraction procedures, and analytical framework prior to commencing the literature search. The complete protocol is available from the corresponding author upon reasonable request. We acknowledge that prospective registration would have further enhanced methodological transparency, and this represents a limitation of the current study. An initial pool of 447 records was identified through systematic searches across PubMed (https://pubmed.ncbi.nlm.nih.gov/), Scopus (https://www.scopus.com/), Web of Science (https://www.webofscience.com/), and PsycINFO (https://www.apa.org/pubs/databases/psycinfo) databases. After the initial screening process:

• 198 duplicate records were removed.

• 23 non-English language studies were excluded.

• 36 records were excluded for being published before 2020.

• 65 records were excluded based on irrelevant titles or non-P300 ERP focus.

This resulted in 125 studies eligible for full-text review. A total of 52 studies were identified as being suitable for inclusion in this systematic review after the inclusion criteria had been applied to include only those studies that are based upon randomized and controlled study designs which utilize clinical populations; these studies have been organized into a comprehensive database format to document study aims, paradigms and measurements used to obtain P300 data, demographic information regarding the participants, specific clinical population(s) studied, type of interventions utilized and study outcomes related to each of the six research questions.

The 52 studies identified were all empirical and employed either a randomized controlled trial (RCT), a controlled clinical trial, or a quasi-experimental design with an appropriately matched comparison group. In addition, the clinical populations examined in these studies included dementia spectrum disorders (Alzheimer’s disease, vascular cognitive impairment, mild cognitive impairment), acquired brain injuries and disorders of consciousness, mood and anxiety disorders, neurodevelopmental and attention disorders, psychotic disorders and addiction, and chronic neurological and medical conditions.

**Fig. 1.**
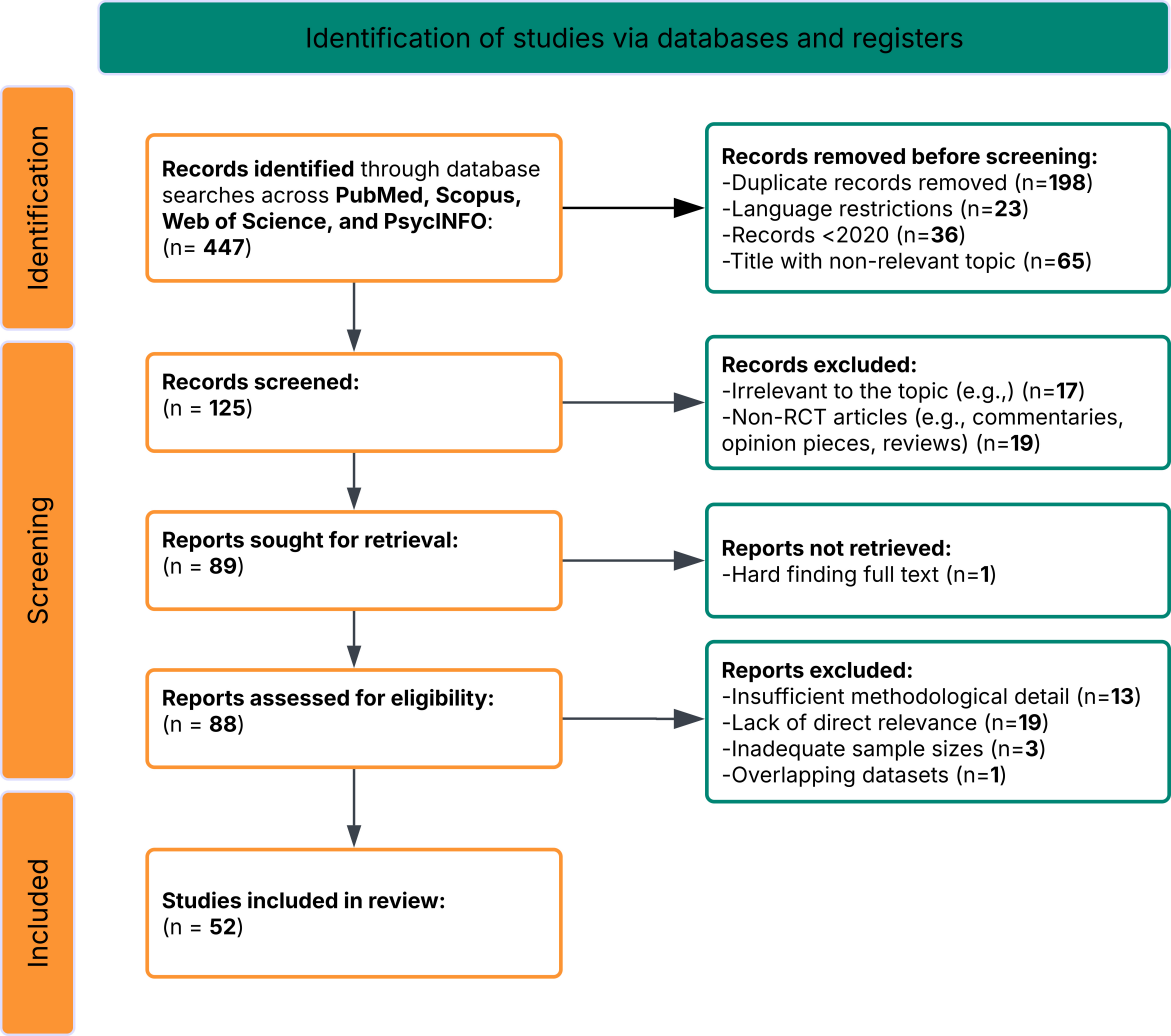
**PRISMA flow diagram of study selection process**. RCT, randomized controlled trial; PRISMA, Preferred Reporting Items for Systematic Reviews and Meta-Analyses.

Additionally, P300 data from the studies were used to examine the relationships between cognitive processing (i.e., attention, context updating, working memory, and cognitive control) and symptomatology, disease progression, treatment response, and functional outcomes in the respective clinical populations. Finally, the results of these studies were synthesized qualitatively to identify consistent, reliable, and clinically applicable biomarkers for addressing each of the six core research questions; the systematic review process is summarized in Fig. 1.

### 2.2 Search Strategy

The search strategy was designed to capture studies examining P300 event-related potentials as cognitive biomarkers in neurological and neuropsychiatric populations. Key search terms included:

• “P300” OR “P3” OR “Event-Related Potentials” OR “ERP” OR “Electroencephalography”.

• “Cognitive Biomarker” OR “Neural Marker” OR “Electrophysiological Marker”.

• “Neurological Disorders” OR “Neuropsychiatric Disorders” OR “Psychiatric Disorders”.

• “Alzheimer’s Disease” OR “Dementia” OR “Mild Cognitive Impairment” OR “Vascular Cognitive Impairment”.

• “Traumatic Brain Injury” OR “Disorders of Consciousness” OR “Stroke” OR “Brain Injury”.

• “Depression” OR “Anxiety” OR “Schizophrenia” OR “ADHD” OR “Addiction”.

• “Treatment Response” OR “Clinical Outcome” OR “Diagnosis” OR “Prognosis”.

• “Randomized Controlled Trial” OR “Controlled Study” OR “Clinical Trial”.

Search strings were adapted for each database to ensure comprehensive coverage:

(“P300” OR “P3” OR “Event-Related Potentials”) AND (“Cognitive Biomarker” OR “Neural Marker”) AND (“Neurological Disorders” OR “Neuropsychiatric Disorders”) AND (“Dementia” OR “Brain Injury” OR “Depression” OR “Schizophrenia” OR “ADHD” OR “Addiction”) AND (“Treatment Response” OR “Diagnosis” OR “Prognosis”) AND (“Randomized” OR “Controlled Trial”).

The search was limited to peer-reviewed articles published in English between January 2020 and August 2025. Only studies reporting empirical P300 ERP data related to cognitive function or clinical outcomes in neurological and neuropsychiatric populations with randomized or controlled study designs were included.

### 2.3 Eligibility Criteria

A structured set of inclusion and exclusion criteria was applied during the screening and selection process to ensure the relevance, rigor, and applicability of the included studies to the research questions.

#### 2.3.1 Inclusion Criteria

• Empirical studies investigating P300 event-related potentials as biomarkers of cognitive function in individuals with neurological or neuropsychiatric disorders.

• Studies utilizing RCT, controlled clinical trial, or quasi-experimental designs with appropriate comparison groups (healthy controls or active/placebo comparators).

• Research examining P300 amplitude, latency, or topographical distribution in relation to clinical variables such as diagnosis, symptom severity, disease progression, treatment response, or functional outcomes.

• Studies involving clinical populations, including dementia spectrum disorders, acquired brain injury, disorders of consciousness, mood disorders, anxiety disorders, psychotic disorders, addiction, neurodevelopmental disorders, and chronic neurological conditions.

• Studies employing standardized P300 elicitation paradigms (auditory oddball, visual oddball, or variant paradigms) with documented ERP acquisition and analysis methods.

• Studies published in peer-reviewed journals between January 2020 and August 2025.

• Articles written in English with full-text availability.

#### 2.3.2 Exclusion Criteria

• Review articles, meta-analyses, editorials, opinion pieces, case reports, or theoretical papers.

• Studies not specifically measuring P300 ERP components or reporting only other ERP markers (e.g., N100, N200, mismatch negativity [MMN]) without P300 data.

• Research focused solely on healthy populations without clinical diagnostic relevance or comparison to patient groups.

• Studies lacking randomized or controlled designs (e.g., uncontrolled observational studies, single-arm trials without baseline comparison).

• Studies published in languages other than English or lacking full-text access.

• Insufficient methodological detail regarding P300 acquisition parameters, analysis methods, or unclear relevance to the defined research questions.

These criteria were systematically applied to refine the evidence base for this review, ensuring that included studies meaningfully contribute to understanding the role of P300-based cognitive biomarkers in neurological and neuropsychiatric research and clinical applications.

### 2.4 Risk of Bias Assessment

Bias in the 52 reviewed studies was assessed using an adapted form of the Cochrane Risk of Bias Tool (RoB 2.0; Cochrane Collaboration, London, UK; https://www.riskofbias.info/), designed to assess risk of bias in neuroimaging research across both clinical and cognitive neuroscience. This version was developed based on the design-specific methodological features of P300 ERP studies, including randomized controlled trials, controlled clinical trials, and quasi-experimental study designs. Six domains were assessed:

(1) Selection Bias (Random sequence generation and allocation concealment)

Low Risk: Randomized controlled trials using randomized or appropriately matched groups were common.

Moderate Risk: Quasi-experimental studies lacking a description of the process by which subjects were assigned to either group or how participants were matched within a study.

(2) Performance Bias (Blinding of participants and personnel)

High to Moderate Risk: Studies utilizing ERP or treatment-based studies with behavioral treatments, neurofeedback, cognitive rehabilitation, or neuromodulation (transcranial direct current stimulation [tDCS]/repetitive transcranial magnetic stimulation [rTMS]) often utilized unblinded participants and personnel.

(3) Detection Bias (Blinding of outcome assessors)

Low Risk: Most studies used objective P300-derived measures (amplitude/latency), standardized clinical assessment tools, and/or automated processing of ERP signals to minimize assessor bias. Although several studies failed to describe assessor-blinding protocols for measuring behavioral outcomes.

(4) Attrition Bias (Incomplete outcome data)

Moderate Risk: Longitudinal intervention studies often reported high dropout rates. While many studies employed statistical methodologies (intention-to-treat analysis/multiple imputation) to address missing data, few provided specific methodological descriptions.

(5) Reporting Bias (Selective reporting of outcomes)

Low Risk: While most studies clearly reported primary P300 and clinical outcomes, a small number omitted secondary results or exploratory analyses, potentially indicating selective reporting.

(6) Other Bias (Funding sources and potential conflicts of interest)

Moderate Risk: Several studies, especially those utilizing commercial EEG equipment, neurofeedback platforms, pharmaceutical interventions, or neuromodulation devices, failed to provide full disclosure of potential conflict of interest or funding influences.

**Fig. 2.**
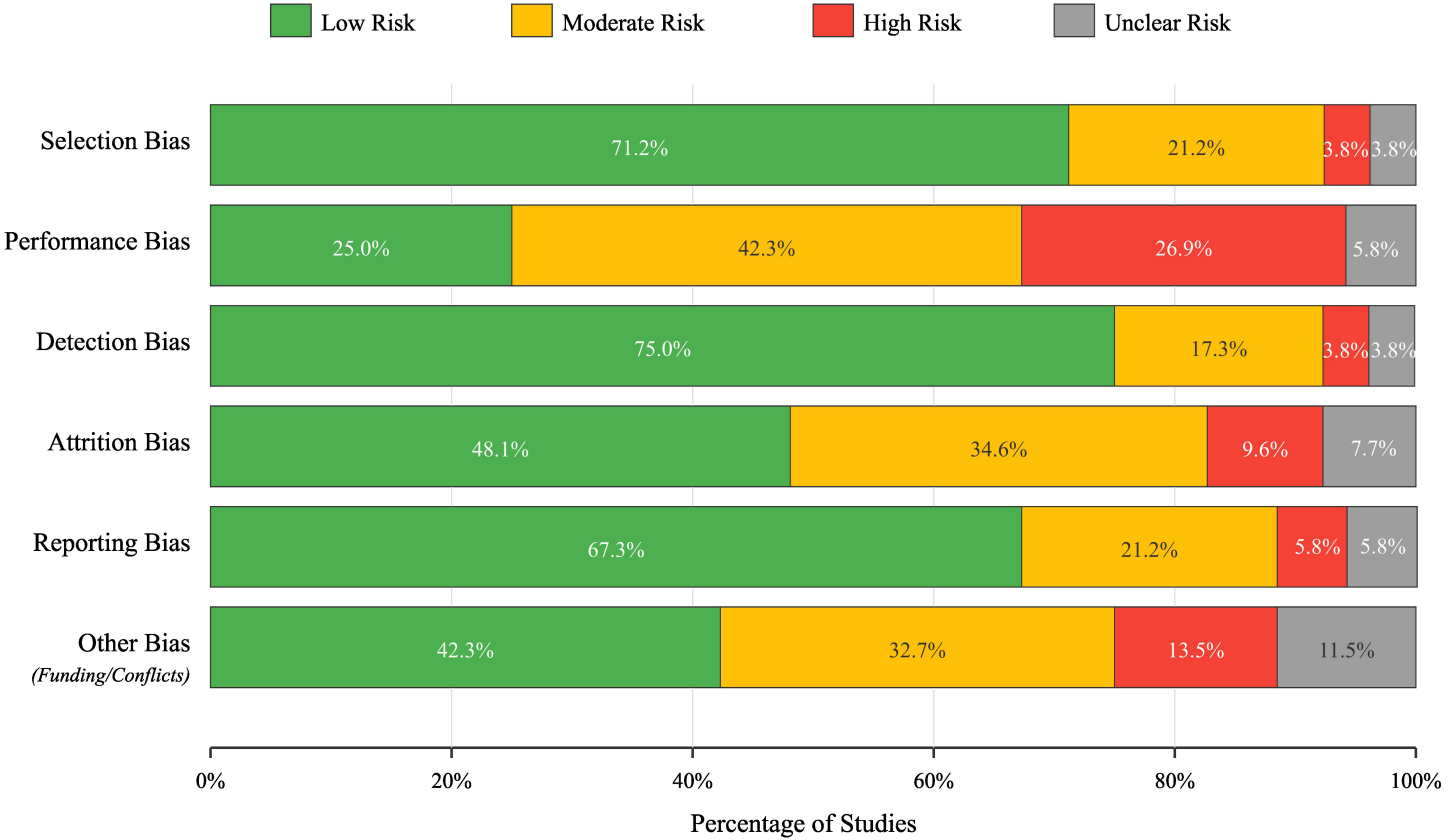
**Risk of bias assessment across 52 studies**.

Two independent reviewers assessed each study within each domain. When two reviewers disagreed on their assessment of a study, they discussed it until they reached an agreement. If the two reviewers could not reach an agreement by discussing the study, a third reviewer would provide their opinion. This process of three reviewers evaluating studies increased the objectivity, clarity, and consistency of their assessments and enhanced the credibility of their study-quality ratings. Overall, across all studies reviewed, the risk of bias ranged from Low to Moderate. It can be stated specifically that the Selection Bias and Detection Bias domains were strong. The strength of these domains was based upon the design of the studies being Randomized Controlled Studies, and the objective nature of the P300-based outcome measure. Thus, the risk-of-bias assessment identified variability across the six domains of the studies reviewed. Therefore, caution is warranted when evaluating the results of studies with unclear blinding practices, incomplete data, or unidentified commercial interests. The summary of the risk-of-bias assessment for all 52 studies is shown in Table 1 and visually represented in Fig. 2.

**Table 1.** **Distribution of risk of bias assessment across 52 included studies**.

Bias domain	Low risk (%)	Moderate risk (%)	High risk (%)	Unclear risk (%)
Selection bias	71.2	21.2	3.8	3.8
Performance bias	25.0	42.3	26.9	5.8
Detection bias	75.0	17.4	3.8	3.8
Attrition bias	48.1	34.6	9.6	7.7
Reporting bias	67.3	21.1	5.8	5.8
Other bias (funding/conflicts)	42.3	32.7	13.5	11.5

The risk-of-bias assessment illustrated the variety of study features across the six studied domains. More specifically, selection bias was generally managed appropriately; approximately 71.2% of studies used either proper randomization or participant matching. Similarly, the detection bias domain also had a low risk of bias (approximately 75%) in most studies; this reflected the inherent objectivity of P300-derived outcome measures and the standardization of ERP analysis pipelines. However, performance bias was much more difficult to manage. Only 25% of the studies were classified as low risk of performance bias, primarily due to the practical constraints of blinding participants and personnel in treatment-based interventions that use neuromodulation, neurofeedback, or cognitive rehabilitation protocols.

Attrition bias showed mixed results: 48.1% of studies demonstrated low risk through complete outcome reporting and appropriate handling of missing data, while 34.6% had moderate concerns regarding dropout rates or unclear handling of incomplete datasets in longitudinal intervention studies. Reporting bias was generally well-controlled (67.3% low risk), though 5.8% of studies showed evidence of selective outcome reporting.

The “other bias” category was of particular importance because it focused primarily on whether there was a conflict of interest or what funded the research. In only about 43 percent of the studies, we were able to classify them as low risk. It is worth noting that almost 14 percent of studies were classified as having a high risk of “other bias” due to commercial affiliations (e.g., EEG equipment manufacturers, pharmaceutical sponsors, neuromodulation device companies), which could have affected how the research was conducted and reported.

Additionally, just over 11 percent of studies did not provide enough information for us to evaluate their risk. These findings suggest that although the overall methodology of the included studies was sufficient—largely because they were randomized, controlled, etc.—the three categories of performance blinding, commercial influence, and drop-out management are important to consider when evaluating their results. There was no doubt that the greatest confidence would be placed on studies that completely documented their methodologies and had zero risk across all categories; that represents less than one-third of the total number of studies.

### 2.5 Data Extraction and Synthesis

The data extraction process was conducted systematically using an extraction form created specifically for this literature review (**Supplementary Table 1** provides complete study characteristics, including population, design, and intervention details). Data extracted from each of the studies included in this literature review were: bibliographical information (author(s), year, publication title), study design features (randomization method, type of control group, use of blinded methodologies), sample features (size of sample, age of participants, gender distribution of participants, clinical diagnoses of participants, diagnostic criteria utilized), P300 paradigm characteristics (type of stimulus used, type of oddball paradigm used, task parameters), P300 recording parameters (electrode placement, sampling frequency, reference), P300 outcome variables (P300 amplitude, P300 latency, electrode site), clinical outcome variables (cognitive assessments, symptom rating scales, functional measures), intervention characteristics (pharmacologic interventions, neuromodulation interventions, rehabilitation interventions) and primary findings related to P300 as a biomarker.

Articles were grouped according to the six research questions and qualitatively synthesized for each group. A summary of the 52 research articles examined in the systematic analysis is presented in Table 2 (Ref. [51, 52, 53, 54, 55, 56, 57, 58, 59, 60, 61, 62, 63, 64, 65, 66, 67, 68, 69, 70, 71, 72, 73, 74, 75, 76, 77, 78, 79, 80, 81, 82, 83, 84, 85, 86, 87, 88, 89, 90, 91, 92, 93, 94, 95, 96, 97, 98, 99, 100, 101, 102]) to highlight the diverse array of P300-based studies examining neurological and neuropsychiatric disorders. The distribution of the studies across research questions was: RQ1 (Dementia Spectrum Disorders) = 11 studies; RQ2 (Acquired Brain Injury and Disorders of Consciousness) = 10 studies; RQ3 (Mood, Anxiety and Stress-Related Disorders) = 7 studies; RQ4 (Neurodevelopmental and Attention Disorders) = 6 studies; RQ5 (Psychotic Disorders and Addiction) = 12 studies; RQ6 (Chronic Neurological and Medical Conditions) = 6 studies.

**Table 2.** **Research articles of systematic analysis (n = 52)**.

Ref ID	Authors (year)	Sample	Methodology	Main findings
**RQ1: Dementia spectrum disorders (n = 11)**				
[51]	Bae J *et al*. (2024)	Age: 54 to 90 years - Sex: HC group - 523 males, 446 females; MCI group - 232 males, 152 females - Cognitive Status: HC group (Cognitively Normal [CN] and Subjective Cognitive Decline [SCD]), MCI group (amnestic MCI [aMCI] and non-amnestic MCI [naMCI]) - Cognitive Function Scores: Lower SNSB II and K-MMSE scores in MCI group compared to HC group; no significant differences in KDSQ-C, K-GDS, and K-IADL scores.	Participants: 1353 elderly participants from the Gwangju Alzheimer’s and Related Dementia (GARD) cohort in South Korea. The intervention in this study is an active auditory oddball task.	- Greater response time variability and P300 latency variability in the MCI group compared to the HC group. - Loss of synchronization in the beta band for the MCI group in response to standard stimuli. - Absence of event-related desynchronization (ERD).
[52]	Gangemi A *et al*. (2024)	Number of participants: 30 - Diagnosis: Vascular dementia - Age range: 50–80 - Mean age: 71.2 ± 5.6 - Age range of participants: 64 to 78 years - Inclusion criteria: Clinical diagnosis.	Study design: Controlled study with two groups (experimental and control) - Intervention: Anodal tDCS for experimental; Transcranial direct current stimulation (tDCS) applied to the dorsolateral prefrontal cortex (DLPFC) for 20 minutes per day over two consecutive weeks.	- Reduction in P300 latency, indicating faster cognitive processing. - Increase in P300 amplitude, suggesting a stronger neural response to cognitive stimuli. - Significant improvement in MMSE scores, indicating overall cognitive function enhancement.
[53]	Hu Y *et al*. (2021)	60 patients with non-dementia vascular cognitive impairment.	Randomized controlled trial (RCT); brain rehabilitation health care measurement system-assisted cognitive training.	- The brain rehabilitation health care measurement system-assisted cognitive training can effectively improve the cognitive function of patients with vascular cognitive impairment and their daily activities.
[54]	Hu Y *et al*. (2020)	Sixty patients with non-dementia vascular cognitive impairment (VCIND).	RCT; Individualized therapy based on the brain rehabilitation health measurement system.	- Significant improvement in cognitive function as measured by Montreal Cognitive Assessment (MoCA), MMSE, and Barthel Index (BI) scores in the training group compared to the control group.
				- Shorter incubation period and higher amplitude of P300 event-related potentials in the training group compared to the control group.
[55]	Hua X *et al*. (2022)	Healthy young males: n = 48, age = 33.4 ± 6.3 years - Healthy elderly: n = 29, age = 63.8 ± 4.0 years - Alzheimer’s disease subjects: n = 11, age = 69.2 ± 7.1 years.	Randomized, double-blind, placebo-controlled phase I trial with single ascending dose, multiple ascending dose, and fixed; Fosgonimeton (ATH-1017).	- Fosgonimeton was safe and well-tolerated across all doses. - Pharmacokinetic results were dose-proportional with no sex effect or accumulation over 9 days. - The main effect on quantitative electroencephalography (qEEG) was acute and sustained gamma power induction. - Significant effect toward ERP P300 latency normalization in AD subjects compared to placebo (*p* = 0.027). Fast-onset normalization of ERP P300 latency suggests enhancement of synaptic function and potential procognitive effects.
[56]	Wang Y *et al*. (2022)	Population size: 60 patients - Condition: Post-stroke cognitive impairment (PSCI) - Study design: RCT.	RCT; Transcranial ultrasound stimulation (TUS) combined with conventional cognitive.	- Significant improvements in cognitive measures (Mini-Mental State Exam, modified barthel index (MBI) score, P300 latency, and wave amplitude) and BDNF levels in both groups after treatment, with greater improvements in the TUS group.
				- Higher scores in executive function, nomination, attention, language, and delayed recall in the TUS group compared to the control group. TUS combined with cognitive rehabilitation was more effective than cognitive rehabilitation alone in improving PSCI. Mechanism may involve upregulation of BDNF and changes in P300.
[57]	Xie W *et al*. (2023)	Recruitment location: Rehabilitation Medicine Center, West China Hospital, Sichuan University, Chengdu, Sichuan Province, China - Age: Participants should not be over 80 years old.	Study type: Single-centre, assessor-blind, randomized controlled clinical trial - Number of arms: Four parallel arms - Control group: Conventional treatment (CT) only - HBOT group: HBOT (2.0 ATA, 40 sessions, 110 min per session, twice daily for 5 days per week for 4 weeks) plus CT. - rTMS group: rTMS (10 Hz, 20 min per session, once daily for 5 days per week for 4 weeks) plus CT. - HBOT combined with rTMS group: HBOT and rTMS combined plus CT.	Primary endpoint: MoCA score - Secondary endpoints: MMSE score - MBI score - P300 latency and amplitude - Cerebral cortical oxygenated and deoxygenated haemoglobin (HBO_2_ and HbR) concentrations as measured by task-state functional near-infrared spectroscopy (fNIRS).
[58]	Yan Y *et al*. (2022)	Total participants: 88 - Location: Fujian Provincial Hospital, China - Recruitment strategies: Memory clinic referrals, electronic health records, community outreach, media outreach.	Single-center, parallel-arm, randomized controlled trial with allocation concealment and outcome assessor blinding. The intervention is a single-stage integral art-based cognitive intervention (SIACI).	Study protocol; results pending completion.
[59]	Zhang J *et al*. (2025)	Clinical population with a relevant diagnosis.	RCT; The intervention is the combination of repetitive transcranial magnetic stimulation.	Study protocol; results pending completion.
[60]	Zhao J *et al*. (2020)	Total participants: 36 - Population: Older individuals with MCI - Group distribution: 18 in the creative expression program (CrExp) group, 18 in the CrExp group, 18 in the control group (CG)	Randomized controlled clinical trial; 16-week CrExp.	- Significant differences in P300 latency between the creative expression group and the control group at post-intervention and follow-up. - Shortened task reaction time in the creative expression group compared to the control group at post-intervention.
[61]	Zhong X *et al*. (2021)	Condition: Cognitive impairment related to cerebral small vessel disease (CSVD) - Number of participants: 106 - Allocation: Randomized into Tai chi exercise intervention group.	Randomized controlled trial with two parallel groups in a 1:1 allocation ratio with allocation concealment and assessor; Tai chi exercise.	Study protocol; results pending completion.
**RQ2: Acquired brain injury & DOC (n = 10)**				
[62]	Bonanno M *et al*. (2025)	Total number of patients: 20 - Condition: Minimally conscious state (MCS) - Group distribution: Experimental group = 10, Control group = 10.	Quasi-randomized clinical trial with an experimental group and a control group; The intervention is the combination of advanced sensory stimulation using the Neurowave system and gentle touch stimulation.	The main findings include a significant interaction effect on P300 latency, indicating that gentle touch stimulation significantly influenced cognitive processing in patients with a minimally conscious state. The study supports the integration of tactile interventions into neurorehabilitation programs, showing significant effects on neurophysiological and functional measures.
[63]	Feng X *et al*. (2025)	Population size: 60 patients - Medical condition: Post-stroke cognitive impairment - Demographic and baseline characteristics: - Age: Median age for control group was 64.5 years.	Double-blind, randomized, controlled trial; Computer-assisted cognitive training (CACT) using the Flex Table digital occupational therapy (OT) equipment.	Both groups showed significant improvements in MMSE, MBI, P300 amplitude, and P300 latency, with the intervention group demonstrating more pronounced improvements. - The intervention group exhibited greater improvements in TMT-A and TMT-B.
[64]	Gangemi A *et al*. (2024)	Total number of participants: 24 - Condition: Chronic MCS - Cause of condition: Vascular or traumatic brain injury - Study location: Neurorehabilitation.	Quasi-randomized controlled study; - Experimental Group: Combined tDCS and Robotic Verticalization Therapy (RVT) - Control Group: RVT alone.	The combination of tDCS with RVT leads to greater improvements in cognitive functioning and a reduction in P300 latency compared with RVT alone in individuals with MCS.
[65]	Gangemi A *et al*. (2025)	Total participants: 28 - Experimental group: 14 - Control group: 14 - Mean age: 58.13 years (SD = 8.33) - Location: U.O.C. Neurorehabilitation Unit of IRCCS Neurolesi “Bonino-Pulejo” in Messina, Italy	Quasi-randomized controlled trial with an experimental group receiving bilateral tDCS and a control group receiving sham; Bilateral tDCS applied to the right and left DLPFC with parameters: 2 mA intensity, 2.5 mA/cm^2^ current density, administered 5 times a week for 4 weeks, totaling 20 sessions.	- Significant improvement in ERP latencies (N200 and P300) indicating enhanced neural responsiveness to cognitive stimuli. - Increased beta band rhythms associated with cognitive functions like attention and memory. - Significant improvements in clinical measures of awareness and functional capacity as assessed by CRS-R, LCF, and FIM. Bilateral tDCS is a potentially effective therapeutic strategy for enhancing awareness and functional abilities in MCS patients.
[66]	Jiang T *et al*. (2023)	Total participants: 38 - Participants receiving real intermittent theta burst stimulation (iTBS): 19 - Participants receiving sham iTBS: 19 - Population: Patients with non-spatial attention deficits.	Prospective, double-blinded, single-center, randomized controlled trial with sham control; iTBS over the left dorsolateral prefrontal cortex.	- Study protocol; results pending completion.
[67]	Li K *et al*. (2024)	Population size: 30 - Condition: Post-stroke comorbid cognitive impairment and depression (PSCCID) - Study design: Randomized into intervention and control groups.	RCT; Repetitive transcranial magnetic stimulation (rTMS) over the left DLPFC for 4 weeks.	- Improvements in cognition, depression status, and neural electrophysiology in both intervention and control groups, with more significant improvements in the intervention group.
				- Enhanced functional connectivity within the default mode network (DMN) in the intervention group compared to pre-intervention and the control group.
				- Positive correlation between DMN connectivity and MMSE scores, and some correlations with P300 latency and amplitude.
				- rTMS is an effective treatment for improving cognitive impairment and depression in PSCCID patients.
				- Enhanced DMN connectivity may serve as a compensatory mechanism for clinical recovery.
[68]	Liu C *et al*. (2024)	Population: Post-stroke patients with cognitive dysfunction - Sample size: 39 participants (29 completed the training).	Randomized Controlled Trial; - Sequential exercise-cognitive training - Simultaneous exercise-cognitive training.	- No significant difference in d-prime between groups.
				- Significant improvements in P300 and theta coherence in the simultaneous training group.
				- Significant enhancements in physical function in the simultaneous training group.
				- Improvements in cognition and multifaceted domains in the simultaneous training group.
				- Potential of technology-driven, personalized rehabilitation for post-stroke patients.
[69]	Quera Salva MA *et al*. (2020)	Adult patients with severe traumatic brain injury (TBI) and significant fatigue symptoms (FSS score ≥4, ESS score ≥10, and/or PSQI >5).	Randomized controlled trial; Blue-enriched white light (BWL) therapy: 30-minute exposure to waking white light enriched with blue for 4 weeks.	- Significant improvement in Fatigue Severity Scale (FSS) score in the BWL therapy group compared to the no-light therapy group.
				- Assessment of the latency change of the P300 component of event-related potentials before and after therapy.
[70]	Wan X *et al*. (2024)	Total number of patients: 26 - Groups: rTMS (n = 13), sham (n = 13) - Recruitment location: Department of Rehabilitation Medicine, Xuan Wu Hospital, Beijing, China	RCT with a single-blind design, involving 26 patients with prolonged disorders of conscious; Parietal rTMS administered at 10 Hz for 10 sessions.	- Significant increase in CRS-R scores in the rTMS group, indicating improved consciousness levels. - Appearance of a P300 waveform in the rTMS group, suggesting restored attention resource allocation abilities and enhanced residual brain functions.
[71]	Xie B *et al*. (2025)	Population: Post-stroke depression patients - Number of participants: 160.	RCT; Phototherapy (daily 40 min).	- Phototherapy significantly increased serum tetrahydrobiopterin (BH4) levels compared to controls.
				- Improved ERP parameters: higher mismatch negativity (MMN) latency, P300 latency, and amplitudes in the phototherapy group.
				- Decreased depressive symptoms: HAMD scores decreased more in the phototherapy group. - Enhanced cognitive function: MoCA scores increased significantly in the phototherapy group. - Reduced inflammation: lower levels of IL-6, TNF-α, and IL-1β in the phototherapy group.
**RQ3: Mood & Anxiety disorders (n = 7)**				
[72]	Desfossés-Vallée S *et al*. (2024)	TS group: 24 participants - OCD group: 18 participants - BFRB group: 16 participants - Control group: 59 participants - Matched on age and nonverbal intelligence.	Participants: TS (n = 24), OCD (n = 18), BFRB (n = 16), Control (n = 59) - Task: Visual counting oddball task - Measure: The intervention is a visual counting oddball task used to elicit Event-Related.	- No group differences for P200 and N200 when controlling for anxiety and depression. - Decrease in the anterior P300 oddball effect for the TS and OCD groups. - Intact oddball effect for BFRB group. - Distinct brain regions involved in the P300 oddball effect.
[73]	Liu H *et al*. (2026)	Age: 12–18 years - Conditions: Depression and recent self-harm (≤2 weeks prior) - Study period: June to December 2024 - Group sizes: rTMS alone (n = 80), rTMS plus group therapy.	Parallel-group RCT; Real-time EEG-triggered rTMS combined with group therapy.	- Greater reduction in Hamilton Depression Rating Scale-24 item (HAMD-24) scores in the observation group (–14.5 ± 3.2 vs. –9.8 ± 2.9, *p * < 0.001).
				- Significant improvements in Hamilton Anxiety Rating Scale-14 item (HAMA-14), Suicide Probability Scale (SPS), and self-harm severity in the observation group.
				- Shorter P300 latency and lower θ/β ratio in the observation group, indicating improved neurophysiological markers.
[74]	Mallorquí-Bagué N *et al*. (2020)	Anorexia Nervosa (AN) group: 20 female patients, mean age = 22.7 years, mean BMI = 16.6 kg/m^2^, 60% restrictive subtype, 40% binge/purging subtype. - Healthy Control (HC) group: 20 female participants, mean age = 21.0 years, mean BMI = 20.7 kg/m^2^. Participants were matched by age and education level.	Cross-sectional study involving a clinical group of patients with anorexia nervosa and a healthy control group, using self-report. The intervention in this study is a computerized task where participants are instructed.	- LPP amplitudes were significantly smaller during the down-regulation of food craving in both groups. - Individuals with AN showed smaller P300 amplitudes compared to healthy controls. - Reappraisal strategies were positively correlated with LPP amplitude.
[75]	Pan N *et al*. (2024)	Number of participants: 66 depressed, 47 healthy controls - Socio-demographic data recorded.	Controlled study design with intervention.	- The theta spectral power of the left frontal lobe was stronger than that of the right in depressed patients, opposite to healthy controls.
				- FTA in F3/F4 affects the emergence of depression and changes in cognitive function.
				- FTA is useful for assessing depression severity and identifying cognitive impairment.
[76]	Santopetro N *et al*. (2020)	Gender: Female - Age Group: Adolescents.	Controlled study design with intervention.	- Reduced baseline P300 amplitude predicts increases in depression symptoms over a two-year period.
				- Baseline P300 is particularly related to increases in anhedonia and negative self-esteem symptoms.
				- Reduced P300 amplitude can be used as a potential risk marker for adolescents at risk of developing increased depressive symptoms.
[77]	Sun Y *et al*. (2023)	Age: 18–60 years - Diagnosis: Major depressive disorder (MDD) with somatic pain - Recruitment: Mental Health Center and Neurobiological Laboratory, Sichuan University, West China.	Randomized controlled trial with two groups: drug therapy (DT) and combined therapy involving rTMS and sertraline. The intervention in this study is repetitive transcranial magnetic stimulation (rTMS) combined with sertraline. The rTMS protocol involved intermittent theta burst stimulation over the left DLPFC with 800 pulses and 1 Hz over the right DLPFC with 800 pulses, administered 5 times a week for 3 weeks.	- Significant improvements in cognitive impairment and pain at 3 weeks in the CT group. - Significant decreases in total depression scores, anxiety scores, and pain scores in the CT group at 6 weeks. - Improvements in P300 and MMN latencies and P300 amplitude in the CT group at 6 weeks. - rTMS combined with antidepressants is effective for rapid symptom improvement in MDD patients with somatic pain.
[78]	White EJ *et al*. (2021)	MDD group: 60 participants, 52% with current major depressive episode, 98% with at least one past major depressive episode, 68% with comorbid anxiety and/or stress disorders.	Study design: Comparative study between MDD and HC groups. - MDD group: Includes participants with and without comorbidities. The interventions in this study are Behavioral Activation (BA) and Exposure Therapy.	- Individuals with MDD exhibited smaller P300 amplitudes than HCs. - Within the MDD group, treatment completers had larger P300 amplitudes than non-completers. - P300 amplitude could predict therapy completion and may be useful in guiding treatment.
**RQ4: Neurodevelopmental & Attention (n = 6)**				
[79]	Barth B *et al*. (2021)	Age: Mean age for slow cortical potential (SCP) = 33.62 ± 10.24 years, fNIRS = 31.24 ± 9.97 years, electromyography (EMG) = 33.65 ± 12.64 years - Sex: SCP = 14 males, 12 females; fNIRS = 14 males, 7 females; EMG = 14 males, 6 females.	RCT - Participants randomly assigned to SCP-NF, fNIRS-NF, or EMG-BF groups - Total of 67; - Slow Cortical Potential (SCP) Neurofeedback; - Functional Near-Infrared Spectroscopy (fNIRS) Neurofeedback; - Semi-active Electromyography Biofeedback (EMG-BF) Control.	- All three groups (SCP-NF, fNIRS-NF, and EMG-BF) showed significant symptom improvements, suggesting non-specific effects.
				- fNIRS learners showed stronger reductions in ADHD symptoms, particularly impulsivity, compared to SCP non-learners.
				- Some adults with ADHD can learn to regulate SCP amplitudes and prefrontal hemodynamic activity. No significant differences in outcomes between groups when considering the whole sample, but learners showed superior effects. Improvements were stable 6 months after training, indicating long-lasting effects. NF has both non-specific and specific modes of action, particularly when learning occurs.
[80]	Bertoni S *et al*. (2024)	Age: Young adults - Condition: Developmental dyslexia - Number: 20 - Other characteristics: Non-action video game players.	Double-blind randomized controlled trial; The intervention is the combination of action video game training with bilateral transcranial random noise stimulation on the posterior parietal cortex.	- Improvements in temporal attention, word text reading, and pseudoword decoding. - Changes in P300 amplitude brain potential. - Enhancement in temporal attention performance related to pseudoword decoding improvement. - Increased efficiency of visual attention deployment and reshaping of fronto-parietal attentional networks.
[81]	Fietz J *et al*. (2025)	Total sample: 41 male autistic adolescents - Age: 12.00 to 17.11 years - Diagnosis: ASD diagnosed by an experienced clinician using the Autism Diagnostic Observation Schedule or Autism Diagnostic Interview-Revised. - IQ score: Above 70, measured with Wechsler Intelligence Scale for Children - Handedness: Right-handed - Language proficiency: Proficient in German - Exclusion criteria: Severe brain injury, neurological, psychotic, or obsessive-compulsive disorders, previous neurofeedback experience - Medication status: Monitored and maintained constant during study.	Randomized, controlled pre-post-test trial with an experimental group receiving slow cortical potential neurofeedback training. The intervention in this study is slow cortical potential neurofeedback training. The control group received treatment as usual, which included four counseling sessions.	- Significant group × time interaction in P300 latency, with shorter latencies in the SCP neurofeedback group and longer latencies in controls. - Trend toward reduced P300 amplitude in the experimental group. - Changes in late LPP component amplitude linked to reaction time in processing positive emotions.
[82]	Kannen K *et al*. (2022)	Total participants: 20 - Gender: 11 female - Age: Mean = 28.55 years, SD = 8.77 - ADHD subtypes: Combined type (57.89%), Predominantly hyperactive-impulsive type (5.26%), Predominantly inattentive type (36.84%) - Comorbidities: Anxiety disorders (36.84%), Affective disorders (21.05%) - Education: Most had a higher education entrance qualification.	Crossover design: The intervention in the study is the application of transcranial alternating current stimulation (tACS), compared with sham (placebo) stimulation.	- No evidence for enhanced P300 amplitude or low-frequency power increase after tACS compared to sham stimulation. - Significant increase in N700 amplitudes after actual stimulation. - No improvement in neuropsychological performance measures related to attention.
[83]	Li Y *et al*. (2025)	Age: 8–12 years - Diagnosis: ADHD.	Randomized controlled trial; EEG-guided adaptive learning, which includes 8 weeks of EEG-monitored cognitive training.	- Significant reduction in theta/beta ratio (*p * < 0.001)
				- Increase in frontal alpha power (*p * < 0.01)
				- Increase in P300 amplitudes (*p * < 0.001)
				- Improved attention span (*p * < 0.001)
				- Improved impulse control (*p * < 0.01)
				- Improved academic performance in math (*p * < 0.001), reading comprehension (*p * < 0.002), and writing (*p * < 0.001).
[84]	Wang A *et al*. (2025)	Total participants: 26 - Gender: Male - Age: Mean 8.64 years, SD 1.30 years - Age range: 6 to 12 years - ADHD subtypes: ADHD-I (30.8%), ADHD-HI (3.8%), ADHD-C (65.4%).	Participants: 26 male children with ADHD, aged 6 to 12 years. - Intervention: 18 mg/day of oral extended-release methylphenidate (MPH) at a dose of 18 mg/day of oral extended-release for 8 weeks.	- Significant improvements in executive function domains after 8 weeks of MPH treatment.
				- Reduced BRIEF2 scores indicating improvement in inhibition, self-monitoring, shifting, emotional control, initiation, working memory, planning/organization, task monitoring, and material organization. Improved behavioral performance in the Go/NoGo task with shorter correct response times and higher accuracy rates. Reduction in Nogo-P300 latency at Fz, Cz, and Pz electrodes, serving as a neural biomarker for treatment response.
**RQ5: Psychotic disorders & Addiction (n = 12)**				
[85]	Cao H *et al*. (2021)	Alcohol-dependence group: 60 male participants, average age 42.33 ± 7.57 years, average age at first drinking 16.83 ± 2.14 years, average daily drinking 217.7 ± 32.63 g/d, average alcohol dependence years 11.30 ± 6.94 years. Normal control group: 40 male participants, average age 42.03 ± 6.61 years.	Controlled study design with intervention.	- Significant reductions in cognitive function scores for speech, attention, delayed memory, and immediate attention in alcohol-dependent individuals compared to controls. - Prolonged latencies and reduced amplitudes of P200 and P300 in alcohol-dependent.
[86]	Chen J *et al*. (2024)	Total participants: 36 - Gender distribution: 6 males and 6 females in each group - Age: Mean ages of 19.7 ± 0.76, 19.5 ± 1.3, and 19.9 ± 1.68 years - Educational duration: Mean durations of 14.9 ± 1.38, 15.0 ± 1.6, and 15.0 ± 1.4 years. - Inclusion criteria: Absence of physiological, psychological, or neurological disorders; No history of substance abuse; Non-engagement in regular physical activity; SAS-C score not less than 40; Commitment to participate exclusively in the experiment.	Longitudinal intervention comparative analysis design with three groups: control, tDCS, and exergame, involving 36 participants. - tDCS group: bilateral dorsolateral prefrontal cortex stimulation with 2 mA transcranial direct current stimulation twice a week for 20 min each time, lasting for 4 weeks. - Exergames group: cognitive somatosensory game intervention with an intensity of 60–80% VO_2_max. - Control group: pseudo-stimulation and health education.	- All groups showed significant reductions in smartphone addiction scores post-intervention. - Significant improvements in executive control and decision-making abilities were observed. - tDCS showed notable increases in P300 amplitudes and decreases in FRN amplitudes, indicating enhanced cognitive resources and inhibitory control. - tDCS, exergames, and pseudo-stimulation all exhibited significant therapeutic effects on smartphone addiction.
[87]	Feng M & Bai Y (2025)	Total participants: 120 - Diagnosis: Schizophrenia - Experimental group: 60 - Control group: 60.	RCT with an experimental group receiving ink painting art therapy and a control group receiving conventional treatment; Ink painting art therapy, which includes teaching basic painting skills, free subject painting, and group discussion, administered for 12 weeks with 90-minute sessions.	- Significant improvements in emotional stability (ESS) and social cognition (SCQ) in the experimental group. - Increase in P300 amplitudes from 8.3 ± 1.2 to 10.6 ± 1.1 in the experimental group. - No significant changes in the control group. - Significant positive effect on emotional stability, social cognition, and P300 amplitude (*p* <0.001).
[88]	Gilleen J *et al*. (2021)	Total participants: 18 - Age range: 18 to 60 years - Gender: Men and women - Diagnosis: Schizophrenia (DSM-5 criteria) - Cognitive status: Cognitively impaired - Medication: On stable dose of second-generation antipsychotic medication. - Clinical stability: Mean PANSS scores changed by fewer than 6 points across study assessment points.	Study design: Randomized, double-blind, placebo-controlled, crossover design - Participants: 18 patients with schizophrenia; Roflumilast, a phosphodiesterase-4 inhibitor, at doses of 100 µg and 250 µg.	- Roflumilast 250 mg significantly enhanced the amplitude of mismatch negativity (MMN) and working memory-related theta oscillations compared to placebo. - No significant effect on early-stage cognitive markers like 40 Hz ASSR or late-stage markers like 40 Hz ASSR or late-stage markers like P300. - Phosphodiesterase-4 inhibition with roflumilast improves intermediate-stage cognitive processing.
[89]	Liang N *et al*. (2022)	Diagnosis: Schizophrenia (SCZ) with treatment-resistant auditory verbal hallucinations (AVHs) - Sample size: CATS group = 32, CBT group = 33.	Pilot randomized comparative trial; - Intervention 1: Virtual reality-based computer AT system (CATS) - Intervention.	- Significant improvements in AVHs after both CATS and CBT treatments. - Additional improvements in omnipotence beliefs, anxiety symptoms, self-esteem, and quality of life in the CATS group at 12-week follow-up. - No general clinical superiority.
[90]	Liu X *et al*. (2020)	Population size: 30 AD patients, 30 healthy controls - Population composition: All male participants - Ethnic background: Chinese - Clinical vs. non-clinical: AD patients were inpatients; HCs were from the local community.	Longitudinal study with repeated measures; 30 AD patients and 30 healthy controls; P300 evoked by a three-stimulus auditory oddball paradigm at two time points: immediately after the last alcohol intake and after a 4-week abstinence period.	- AD patients showed reduced P3a/3b amplitudes compared to healthy controls. - After 4-week abstinence, P3a/3b amplitudes improved in AD patients but remained lower than those of healthy controls. - No significant differences were observed in P3a and P3b latencies. - Cognitive control deficits in AD are both trait- and state-dependent.
[91]	Murray CH *et al*. (2022)	Adolescents: 18–20 years old, n = 12 (6 males, 6 females) - Adults: 30–40 years old, n = 12 (6 males, 6 females).	Randomized, double-blind, combined within and between-subject design with repeated measures ANOVA (RM-ANOVA) analysis.	- Adolescents are more sensitive to performance-impairing effects of THC, showing dose-dependent impairments in reaction time, response accuracy, and time perception.
				- THC dose-dependently decreases P300 amplitude in adolescents but not in adults.
[92]	Murray CH *et al*. (2022)	Healthy adults.	Within-subjects, double blind design; Δ9-THC (7.5 and 15 mg oral).	- Δ9-THC modulates outcome processing by reducing RewP amplitudes after outcome feedback and, at higher doses, reduces P300 and LPP amplitudes following hits compared to misses, suggesting an “amotivational” state.
[93]	Song Y *et al*. (2025)	Internet Gaming Disorder (IGD): 25 participants, DSM-5 ≥6, ≥14 hours per week gaming - Regular Gaming Use (RGU): 22 participants, DSM-5 <5, ≥14 hours per week gaming - HC: 18 participants, DSM-5 <5, <7 hours per week gaming. tDCS intervention: 46 IGD participants (23 active, 23 sham).	Randomized controlled double-blind study with EEG data collection and tDCS intervention over 2 days (20 minutes each session); tDCS intervention targeting the parietal lobe (Pz) during cue exposure, conducted over two days with 20-minute sessions each, using cathodic stimulation.	- The P300 component in the parieto-occipital lobe is a notable marker for IGD during cue-reactivity tasks. - The P300 component at Pz is particularly influential in distinguishing IGD from other groups. - The Delta, Theta, and Alpha band energies of the P300 component at Pz are positively correlated with current craving in IGD. tDCS intervention targeting Pz during cue exposure significantly reduces craving and game usage time in IGD participants with long-term effects.
[94]	Vollstädt-Klein S *et al*. (2025)	Age: 18 to 65 years - Diagnosis: Alcohol use disorder (AUD) according to DSM-5 criteria - Comorbidities: May include other substance use disorders.	RCT with multiple groups and longitudinal assessments; - Anodal stimulation over right DLPFC - Anodal stimulation over lateral occipital cortex - Sham tDCS - Computerized inhibition training - Treatment as usual (TAU).	Study protocol; results pending completion.
[95]	Xue Y *et al*. (2020)	Participants: 25 male patients with nicotine dependence (ND) and 25 healthy controls - Age: ND group mean age = 32.0 years, HC group mean age = 30.9 years - Education: ND group mean education = 14.2 years, HC group mean education = 15.0 years. - Ethnicity: All participants were Chinese. - Smoking habits: ND group smoked an average of 17.5 cigarettes per day, FTND score = 7.5. - Gender: Only male participants were included in the study.	Study design: Investigate the effects of 2-hour tobacco abstinence on cognitive control in patients with nicotine dependence (ND) using ERP P300 measurements. - Measurements: ERP P300 at normality state (immediately after last cigarette) and abstinence state (2 hours after last cigarette) for ND group; ERP P300 twice with a 2-hour interval for HCs. - Paradigm: Three-stimulus auditory oddball paradigm to evoke ERP P3a and P3b components.	- Significant differences in CO levels between abstinence and normality states in ND group. - No significant differences in HAMD and HAMA scores during abstinence. - Significant cognitive control deficits in ND group compared to healthy controls (reduced P3a and P3b amplitudes, prolonged P3a latency). - 2-h tobacco abstinence has no effect on cognitive control deficits in male patients with ND.
[96]	Yang X *et al*. (2025)	Total participants: 84 - Female participants: 39 (46.43%) - Mean age: 21.09 years - Inclusion criteria: - Played “Honor of Kings” for more than 21 hours per week.	Study Design: RCT Participants: 84 individuals with IGD Groups.	- Repeated closed-loop auditory exposure during slow-wave sleep significantly reduced cravings and playtime in individuals with Internet Gaming Disorder. - This intervention was more effective during sleep than during wakefulness.
**RQ6: Chronic neurological & Medical (n = 6)**				
[97]	Duan H *et al*. (2020)	Patients with stable chronic obstructive pulmonary disease (COPD).	Single-centre randomized controlled trial with assessor and data analyst blinding; - Pulmonary-based Qigong exercise - Elastic band-based resistance exercise (RE); - Combination of pulmonary-based Qigong exercise and elastic band-based RE.	Study protocol.
[98]	Ebenezer A *et al*. (2025)	Total participants: 60 - Age: - Medication-only group: Mean age 50.67 years (SD = 12.88) - VRT + Medication group: Mean age 46.81 years (SD = 12.13) - Health status: Normal.	Randomized control trial with unstratified block randomization, allocation concealment, and blinding. - Medication-only group: Betahistine - VRT + Medication group.	- Significant improvements in cognitive performance in the VRT + Medication group, particularly in digit span and task-switching tests. - Reduced P300 response latency and increased amplitude in the VRT + Medication group.
[99]	Gongora M *et al*. (2020)	Health status: Healthy adults - Sample size: 13 - Handedness: Right-handed.	RCT with a within-subjects design using an oddball paradigm to assess the effects of Levetiracetam (LEV) vs placebo acute administration.	- Main effect of condition on P300 amplitude for frontal, central, and parietal electrodes.
				- Significant differences between electrodes as per post hoc comparisons.
				- Reduction in P300 latency during the LEV condition compared to the placebo.
				- Findings support the neural efficiency hypothesis due to reduced P300 latency.
[100]	Hinojosa-Segura C *et al*. (2022)	Total participants: 89 - Gender: 54 women (60.7%), 35 men (39.3%) - Age: 53 ± 7.9 years - Education: 8.3 ± 3.4 years of schooling.	Before-and-after design with a short-term intervention (3 days) using L-Ornithine and L-Aspartate (LOLA) in patients with m; LOLA 18 grams/3 days, administered as 6 g/3 times.	- Significant improvement in PHES scores after LOLA treatment (*p* < 0.0001). - Significant increase in FCP scores after LOLA treatment (*p* < 0.0001). - Significant reduction in P300 latency after LOLA treatment (*p* = 0.015).
[101]	Linnhoff S *et al*. (2023)	Number of participants: 18 - Type of MS: Relapsing-remitting MS - Inclusion criteria: 3 months post-relapse or corticosteroid use, no upper limb paresis, no other neurological.	Study type: Pseudorandomized, single-blinded, sham-controlled trial - Design: Between-subject design in Phase I, cross; tDCS with anodal stimulation.	- Decrease in subjective trait fatigue ratings lasting at least four weeks after stimulations, but this effect was observed in both anodal and sham groups, suggesting a placebo effect.
				- No significant effects of tDCS on subjective state fatigue.
[102]	Shafiyev J & Karadaş Ö (2024)	Age range: 18 to 50 years - Gender distribution: 50.6% male, 49.4% female - Mean age: 35.4 years (±13) - Number of participants: 300 epilepsy patients, 20 healthy controls - Inclusion criteria: Epilepsy patients without syndromic diagnoses affecting cognitive functions.	Prospective randomized study; The intervention in this study is the administration of antiepileptic drugs (ASMs).	- The study demonstrated the detrimental effects of certain ASMs, particularly topiramate and carbamazepine, on cognitive functions.
				- The negative impact on cognitive performance increased with polytherapy compared to monotherapy.
				- Significant differences were observed in P300 and N200 latencies and N2P3 amplitudes between healthy controls and both monotherapy and polytherapy groups.
				- Levetiracetam (LEV), lamotrigine (LTG), and lacosamide (LCM) did not show significant changes in MoCA scores after three months, while topiramate (TPM) and carbamazepine (CBZ) showed significant decreases.
				- Subgroups with TPM and CBZ had lower MoCA scores, indicating a more negative impact on cognitive functions.

Abbreviations: KDSQ-C, Korean Dementia Screening Questionnaire–Cognition; K-GDS, Korean Geriatric Depression Scale; K-IADL, Korean Instrumental Activities of Daily Living; SNSB II, Seoul Neuropsychological Screening Battery, Second Edition; IQ, Intelligence Quotient; EPR, Event-Related Potentials; AD, Alzheimer’s disease; ADHD, attention-deficit/hyperactivity disorder; ASD, autism spectrum disorder; DOC, disorders of consciousness; HC, healthy controls; MCI, mild cognitive impairment; MS, multiple sclerosis; MMSE, Mini-Mental State Examination; EEG, electroencephalography; BDNF, Brain-Derived Neurotrophic Factor; HBOT, Hyperbaric Oxygen Therapy; TMT, Trail Making Test; CRS-R, Coma Recovery Scale-Revised; HAMD, Hamilton Depression Rating Scale; OCD, Obsessive-Compulsive Disorder; BFRB, Body-Focused Repetitive Behaviors; LPP, late positive potential; FTA, frontal theta asymmetry; SCP-NF, slow cortical potential neurofeedback; fNIRS-NF, functional near-infrared spectroscopy neurofeedback; EMG-BF, electromyography biofeedback; tACS, transcranial alternating current stimulation; CBT, cognitive behavioral therapy; VRT, vestibular rehabilitation therapy; PHES, psychometric hepatic encephalopathy score; FCP, figure connection procedure; LCF, Levels of Cognitive Functioning; FIM, Functional Independence Measure; TNF-α, tumor necrosis factor-alpha; IL, interleukin; Fz, frontal; Cz, central; Pz, parietal midline; VRT, vestibular rehabilitation therapy.

The qualitative synthesis was designed to identify common patterns in P300 results across studies within each diagnostic category, evaluate the strength of evidence supporting the use of P300 as a diagnostic, prognostic, or treatment-response biomarker, and assess the clinical relevance and translational potential of P300-based assessment tools. A meta-analysis was not performed due to substantial heterogeneity in study designs, P300 paradigms, clinical populations, and outcome reporting across the included studies.

### 2.6 Data Synthesis and Analysis

#### 2.6.1 Synthesis Approach

Given the substantial clinical, methodological, and statistical heterogeneity across included studies—including variations in diagnostic populations, P300 paradigms, electrode configurations, preprocessing pipelines, intervention types, and outcome reporting—a primarily narrative synthesis approach was adopted. This decision was made a priori based on anticipated heterogeneity that would limit the interpretability of pooled effect estimates.

#### 2.6.2 Quantitative Synthesis

Where sufficient homogeneity existed within diagnostic categories or intervention types, random-effects meta-analyses were conducted using the restricted maximum likelihood (REML) estimator. Heterogeneity was assessed using Cochran’s *Q* statistic, I^2^ percentage, and τ^2^ (between-study variance). I^2^ values were interpreted as low (<25%), moderate (25–75%), or high (>75%) heterogeneity. Prediction intervals were calculated to estimate the range of true effects in future studies. Subgroup analyses were conducted by intervention type and diagnostic category where feasible.

#### 2.6.3 Sensitivity Analysis

Sensitivity analyses were conducted, excluding studies rated as high risk of bias (*n* = 5) to assess the robustness of main findings. Leave-one-out analyses were performed for quantitative syntheses to identify influential studies.

#### 2.6.4 Publication Bias Assessment

For meta-analyses including 10 or more studies, publication bias was assessed through visual inspection of funnel plot asymmetry and Egger’s regression test for small-study effects. The potential impact of publication bias is discussed in the context of the broader limitations of the evidence base.

#### 2.6.5 Certainty of Evidence

The overall certainty of evidence for key findings was assessed qualitatively using principles from the Grading of Recommendations, Assessment, Development, and Evaluations (GRADE) framework, considering risk of bias, inconsistency, indirectness, imprecision, and publication bias. Evidence certainty was rated as high, moderate, low, or very low for primary conclusions. Formal GRADE assessment was not conducted due to the heterogeneous nature of outcomes and the predominantly narrative synthesis; this represents a limitation acknowledged in the Discussion.

## 3. Results

The results of this systematic review synthesize findings from 52 randomized and controlled studies examining P300 event-related potentials as cognitive biomarkers across neurological and neuropsychiatric disorders. The included studies explored diverse clinical populations encompassing dementia spectrum disorders, acquired brain injury and disorders of consciousness, mood and anxiety disorders, neurodevelopmental conditions, psychotic disorders and addiction, and chronic neurological and medical conditions. Studies employed various P300 elicitation paradigms—predominantly auditory and visual oddball tasks—to assess neural correlates of attention, cognitive processing, and treatment response.

This section is organized around the six core research questions, presenting thematic insights into P300’s diagnostic utility, prognostic value, and sensitivity to therapeutic interventions. The findings reveal consistent patterns of P300 abnormalities across disorders—particularly prolonged latency and reduced amplitude—and highlight disorder-specific manifestations that support differential assessment. Attention is given to the relationship between P300 parameters and clinical outcomes, the effects of pharmacological and neuromodulation interventions on P300 normalization, and the translational potential of P300 biomarkers for clinical implementation. The results section focuses on the intersection of neural activity, clinical relevance, and translational applicability, highlighting P300’s unique position as an objective, temporally precise marker that bridges neurophysiological assessment and clinical care across the spectrum of neurological and neuropsychiatric conditions.

### 3.1 [RQ1] P300 as Cognitive Biomarker in Dementia Spectrum Disorders

Eleven studies examined P300 as a cognitive biomarker for diagnosis, progression monitoring, and treatment response in dementia spectrum disorders, including AD, VCI, vascular dementia (VaD), and MCI. The evidence consistently demonstrates that P300 abnormalities—particularly prolonged latency and reduced amplitude—serve as sensitive indicators of cognitive dysfunction in these populations, with significant potential for monitoring the effects of interventions.

#### 3.1.1 P300 Latency as a Marker of Cognitive Processing Speed 

In multiple studies on the entire range of dementia, P300 Latency is emerging as one of the most consistent biomarkers. Researchers in their study [51], using a two-channel portable EEG device, tested 1754 elderly participants in the Gwangju Alzheimer’s and Related Dementia (GARD) cohort for greater P300 Latency variability in MCI than in their normal-aging counterparts. The researchers found that in the MCI group, decreased beta-band synchrony and event-related desynchronization (ERD) in response to stimuli indicated reduced task preparation and impaired performance. The highest variability in coherence values was seen in the amnestic type of MCI; these findings indicate that decreased neural synchrony is an earlier indicator of cognitive deterioration. Other researchers [60] conducted a randomized clinical trial in 36 older adults with MCI, including a 16-week creative expression intervention that significantly improved P300 Latency compared with those who received no treatment. Moreover, the interventions had a sustained positive effect on P300 Latency when the participants were retested after the intervention ended. While the P300 Amplitude was significantly different between the intervention and control groups, there was no difference in the P300 Amplitude values between the two groups; therefore, it appears that P300 Latency is more responsive to treatments in the MCI population. Likewise, all of the studies examining vascular cognitive impairment have reported that P300 Latency has been significantly improved by treatment. Also, researchers in their study [56] reported that 60 patients with post-stroke cognitive impairment who underwent transcranial ultrasound stimulation (TUS) in conjunction with cognitive rehabilitation had significantly improved P300 latency and increased Brain-Derived Neurotrophic Factor (BDNF) levels, suggesting that changes in cognition are due to neuroplasticity.

#### 3.1.2 P300 Amplitude as a Marker of Neural Resource Allocation 

Research studies have shown that the peak amplitude of the P300 is responsive to cognitive rehabilitation methods. A study [52] demonstrated a significant increase in P300 peak amplitude in 30 patients diagnosed with vascular dementia after receiving tDCS to the left Dorsolateral Prefrontal Cortex (DLPFC). The quasi-randomized controlled study also showed significantly reduced P300 latencies (faster cognitive processing), increased P300 amplitudes (greater neuronal activation in response to cognitive stimuli), and clinically meaningful improvements in their Mini-Mental State Examination (MMSE) scores. This suggests that tDCS may be an effective therapeutic method for improving cognitive performance in individuals diagnosed with vascular dementia. Two other studies [53, 54] demonstrated that participants receiving individually tailored cognitive training using a brain rehabilitation health measurement system had both faster P300 latencies and greater amplitudes than participants without cognitive training who had vascular cognitive impairment (non-dementia VCIND). Each of the two randomized controlled trials included 60 participants per condition. Statistically and clinically significant improvements in Montreal Cognitive Assessment (MoCA), MMSE, and Barthel Index scores were observed in participants who received the training compared with controls. Furthermore, statistically significant improvements in P300 measures were positively related to clinically relevant improvements in cognitive performance. These results further support the concept that the P300 is a neurophysiological measure of treatment efficacy that correlates with improved cognitive performance.

#### 3.1.3 Pharmacological Effects on P300 in Alzheimer’s Disease 

A Phase I randomized, double-blind, placebo-controlled study [55] examined a group of 48 young male, 29 older male, and 11 middle-aged male subjects with mild-to-moderate AD, all receiving single ascending doses of ATH-1017 (fosgonimeton), a hepatocyte growth factor/mesenchymal-epithelial transition factor (HGF/MET) positive modulator. Results of this study demonstrated that AD subjects treated with ATH-1017 exhibited a reduction in P300 latency compared with the control group (*p* = 0.027). This treatment produced rapid-onset effects on the subjects’ cognitive function post-dosing. Quantitative electroencephalography demonstrated that ATH-1017 produces both immediate and sustained increases in gamma power post-dosing. These results support the use of P300 as a pharmacodynamic biomarker to identify potential new therapeutics for cognitive enhancement in AD, thereby facilitating early detection of precognitive effects in clinical studies.

#### 3.1.4 Multimodal and Combined Intervention Approaches 

These studies used a combination of interventions and P300 as an outcome measure to assess their effects on cognition. The study [57] was an RCT utilizing a double-blinded assessor design with four parallel groups (control group, Hyperbaric Oxygen Therapy [HBOT]; rTMS alone; and HBOT + rTMS) for individuals aged less than 80 years old with VCI. Secondary outcomes were P300 amplitude and latency; MoCA, MMSE, Modified Barthel Index (MBI), and functional near-infrared spectroscopy (fNIRS)-measured cortical blood flow. This study hypothesized that the combination of HBOT and rTMS would be effective in improving cognitive function by increasing the partial pressure of oxygen, enhancing neuronal excitability, and increasing cerebral cortex activity.

The use of P300 as an outcome measure reflects the growing recognition of its utility in assessing multiple types of therapeutic interventions. The study [58] described a single-stage integral art-based cognitive intervention (SIACI) program for 88 older adults with cognitive impairments; the SIACI program included assessment with both cognitive tests and P300. A second study [61] was an RCT investigating the effects of 8-Style Tai Chi on 106 patients with cognitive decline due to cerebral small vessel disease. A third study [59] proposed using rTMS in conjunction with transcutaneous auricular vagus nerve stimulation (taVNS) to treat MCI, with P300 as the neurophysiological endpoint. Collectively, these studies demonstrated the growing trend in the field toward rigorous investigation of the effectiveness of non-pharmacologic interventions for dementia-related conditions using P300 as a biomarker.

Collectively, these studies provide strong evidence that P300 can be effectively used as a biomarker to assess cognitive status across the dementia spectrum. Furthermore, these studies suggest that P300 has value in measuring treatment response across the dementia spectrum. Specifically, the relationship between improved P300 latency and improved cognitive processing speed provides a potential mechanism for understanding how P300 can be used to monitor treatment efficacy. Additionally, improved P300 amplitudes indicate increased neural resources available for attentional tasks. Therefore, the correlation between improved P300 values and improved clinical cognitive measures (e.g., MMSE, MoCA, Barthel Index) further supports the translational validity of P300 as a biomarker for clinical application. The diverse set of intervention modalities (i.e., cognitive training; tDCS; TUS; pharmacotherapy; and multimodal approaches) that have shown sensitivity to P300 further support the broad applicability of P300 in dementia-related clinical research.

### 3.2 [RQ2] P300 as Cognitive Biomarker in Acquired Brain Injury and Disorders of Consciousness

Ten studies investigated P300 as an effective biomarker for predicting recovery of consciousness, effects of cognitive rehabilitation, and overall functional outcome in patients who suffered from an acquired brain injury, such as post-stroke cognitive impairment (PSCI) and post-stroke depression (PSD); traumatic brain injury (TBI); and a minimally conscious state (MCS). The results provide strong support that P300 is also a valuable prognostic indicator of the individual’s capacity to recover, and serves as a highly sensitive measure of the degree of change associated with interventions intended to promote cognitive improvement—particularly in disorders of consciousness where behavioral assessments are frequently limited.

#### 3.2.1 P300 in Disorders of Consciousness 

Studies demonstrate that the P300 has potential as a tool for monitoring and predicting outcomes in patients with disorders of consciousness (DOC).

Researchers in their study [70] conducted a randomized, double-blind, placebo-controlled trial involving 26 patients with prolonged DOC. They found that applying rTMS at 10 Hz for 10 sessions to the parietal region elicited P300 waveforms in the treatment group. The generation of P300 waveforms in these patients was interpreted as a restoration of their ability to allocate attention resources and as an enhancement of residual brain function. In addition, the authors reported increased potentials in the topographic mapping data from the treatment group (particularly in the left prefrontal cortex) and increased power in the delta and theta frequency bands, indicating increased frontal activity. The authors interpret their findings as evidence that the parietal cortex is a promising target for rTMS in the treatment of prolonged DOC and suggest that this intervention may enhance connectivity between the frontal and parietal regions and improve consciousness recovery in DOC patients.

Researchers in their studies [64, 65] have published several studies on the use of tDCS in patients with MCS. A quasi-randomized controlled study of 24 patients with chronic MCS secondary to either traumatic or vascular injury [64], demonstrated that when tDCS was administered in conjunction with robotic verticalization therapy (RVT) the tDCS/RVT combination resulted in greater decreases in the latency of the P300 than did the RVT-alone condition. The second study by the same author [65] used a similar sample size of MCS patients (n = 28) and demonstrated that bilateral tDCS applied to the DLPFC significantly reduced the latency of the N200 and P300 components of the ERP waveform. The reduced latency of the N200 and P300 waveforms suggests enhanced responsiveness of the patient’s neural systems to cognitive stimuli.

Enhanced beta-band rhythmic activity was also associated with higher-order cognitive processes, such as attention and memory. Clinically, the Coma Recovery Scale-Revised (CRS-R), Levels of Cognitive Functioning (LCF), and Functional Independence Measure (FIM) were all used to measure changes in the level of functioning of MCS patients. As a result of tDCS therapy, significant improvements in clinical measures of awareness and functional capacity were observed in MCS patients. The results of this study support the notion that bilateral tDCS may be a useful therapeutic approach for improving awareness and functional abilities in patients with MCS.

Another group of researchers [62] conducted a quasi-randomized clinical trial involving 20 MCS patients to examine the effects of gentle touch stimulation, delivered in conjunction with the advanced sensory stimulation provided by the Neurowave system. Researchers in the same study [62] reported a significant interaction effect on P300 latency. That is, the authors found that the type of sensory stimulation delivered significantly affected the patient’s cognitive processing. Therefore, they support the inclusion of multimodal tactile interventions in neurorehabilitation programs, with P300 serving as an objective marker of the neural responsiveness to sensory input.

#### 3.2.2 P300 in Post-Stroke Cognitive Impairment 

Studies have shown that P300 can be sensitive to rehabilitation interventions in PSCI. In one such study [63], a double-blind, randomized controlled trial compared to 60 patients who received either computer-assisted cognitive training (CACT) or standard care for 6 months. Each group improved significantly on the modified MMSE, modified barthel index, P300 amplitude, and P300 latency. However, improvements in both the Trail Making Tests (TMT-A, TMT-B) and the auditory verbal learning test-Huashan version (AVLT-H) were much larger in the CACT group, suggesting that CACT improved cognitive function by enhancing attention, executive functions, and memory, with the P300 as a neurophysiological marker of these improvements.

In another study [68], researchers assessed the efficacy of 2 hybrid exercise-cognitive training interventions in 39 post-stroke patients with cognitive dysfunction. A randomized controlled trial was conducted, and the interventions were applied sequentially and in parallel to assess their efficacy. Although the results indicated no significant differences in the d-prime values between the groups, the simultaneous training group had significant improvements in P300 and theta coherence, as well as significant enhancements in physical function. These findings indicate that technology-based, individualized rehabilitation methods can be effective and that the P300 is an indicator of gains in neural efficiency.

Furthermore, researchers in their study [67] investigated the effects of rTMS in 30 patients with post-stroke comorbid cognitive impairment and depression (PSCCID). The patients were assigned randomly to receive either rTMS or sham treatment over the left DLPFC for 4 weeks. Compared to the control group, the rTMS group demonstrated significant improvements in cognition, depression, and neural electrophysiology. Additionally, the rTMS group showed increased functional connections in the default mode network (DMN), which correlated positively with MMSE scores, and there were some correlations between DMN connectivity and P300 latency and amplitude. These findings support the hypothesis that increased DMN connectivity serves as a compensatory mechanism for clinical recovery and that the P300 reflects the effects of rTMS on the neural circuitry.

#### 3.2.3 P300 in Traumatic Brain Injury and Post-Stroke Depression 

The three studies above have utilized the P300 waveform as an electroencephalographic biomarker to assess and quantify changes in the extent of cognitive dysfunction and recovery in patients who have suffered acquired brain injuries such as stroke, TBI, and other conditions resulting in loss of conscious awareness. In the study [69], the use of blue-enriched white light (BWL) was evaluated for its ability to reduce fatigue in adults who had survived severe TBI. While there are a variety of reasons why fatigue is present in many people with acquired brain injury, the most common reason is due to sleep-wake cycle disturbances. Therefore, evaluating the impact of light on sleep-wake cycles in individuals with TBI is relevant. This is especially true given that many commonly used treatments for fatigue include pharmacologic agents that can cause drowsiness (sedatives), which would further contribute to sleep-wake disturbances.

In their evaluation of the effectiveness of BWL therapy, the researchers [69] demonstrated that fatigue symptomatology, as measured by the Fatigue Severity Scale (FSS), was significantly reduced in the BWL group compared with the control group. To evaluate whether improvements in FSS scores reflected objective physiological changes in addition to self-reported symptomatology, researchers also obtained P300 latencies. They reported that while both groups showed a trend towards decreasing P300 latencies, the difference between the two groups was statistically significant at the end of the 2-week study period. Thus, they were able to provide objective measures of improved cognitive performance, in addition to their participants’ subjective ratings of fatigue improvement.

In the study [71], the investigators conducted a randomized controlled trial using a sample of 160 individuals who had experienced a stroke and developed post-stroke depression. In this study, the investigators evaluated the impact of phototherapy on serum tetrahydrobiopterin (BH4) levels and how these changes affected ERPs. The investigators found that phototherapy significantly increased serum BH4 levels compared with controls, which in turn led to improved ERP parameters, including P300 latency and amplitude, in the phototherapy group. In addition to the improvements in ERP parameters, the investigators also observed significant reductions in depressive symptoms (as evidenced by lower Hamilton Depression Rating Scale [HAMD] scores), improved cognitive function (as evidenced by higher MoCA scores), and reduced inflammation (as evidenced by lower interleukin [IL]-6, tumor necrosis factor-alpha [TNF-α], and IL-1β levels).

Based on the results of their study, the investigators proposed a mechanistic pathway linking phototherapy, BH4 metabolism, neuroinflammation, and cognitive improvement, with P300 serving as a biomarker reflecting the neurobiological changes associated with these processes.

Finally, researchers [66] recently published a protocol for a prospective, double-blinded, single-center randomized controlled trial to investigate the use of intermittent theta burst stimulation (iTBS) to improve post-stroke non-spatial attention deficits. The investigators will randomly assign 38 participants to receive either real or sham iTBS over the left DLPFC, along with conventional attention training. P300 has been selected as one of the primary neurophysiologic outcome measures for this study based upon the literature demonstrating its sensitivity to attention-related interventions.

The collective findings of the three studies reviewed above demonstrate that P300 can serve as a useful biomarker for assessing the degree of residual cognitive function in disorders of consciousness, tracking changes in cognition during rehabilitation, and predicting the likelihood of functional recovery in patients with acquired brain injury. Further, the fact that P300 waveforms appear or normalize after neuromodulation interventions (such as tDCS, rTMS, cognitive training, light therapy, and multimodal sensory stimulation) suggests that enhanced cortical processing may occur prior to or in conjunction with clinical improvement. The convergence of evidence from these diverse areas of research further supports P300’s utility as a sensitive biomarker across multiple rehabilitative therapies for acquired brain injury.

### 3.3 [RQ3] P300 as Cognitive Biomarker in Mood, Anxiety, and Stress-Related Disorders

Seven studies used the P300 to determine if it was a cognitive biomarker for the level of symptoms, how well treatments would work, and cognitive impairment in mood and anxiety/stress related disorders, which included: Major Depressive Disorder (MDD), Anxiety Disorders, Obsessive-Compulsive Disorder (OCD), Tourette Syndrome (TS), Body-Focused Repetitive Behaviors (BFRB), and Eating Disorders. The results from these studies support the conclusion that the P300 is useful as both a biomarker of current cognitive function and, uniquely among the disorders studied, a biomarker for predicting future treatment success and symptom progression.

#### 3.3.1 P300 as Treatment Response Predictor in Depression

In their study [78], the researchers found that, for depressed patients participating in a monetary incentive delay task, P300 amplitudes measured at baseline predicted whether patients would complete therapy. They conducted an additional study using data from two clinical trials (60 patients with MDD, 40 healthy controls). Results from this second study indicated that, on average, MDD patients produced P300 amplitudes smaller than those of healthy controls. Importantly, although both groups comprised MDD patients, those who completed therapy had greater baseline P300 amplitudes than those who did not complete therapy—regardless of the type of therapy administered (Behavioral Activation or Exposure Therapy). The authors’ findings suggest P300 can help inform treatment choices as a tool to identify patients who are most likely to participate and successfully complete therapeutic interventions—an innovative use of P300 that extends its utility from diagnosis and/or symptom measurement to treatment planning.

#### 3.3.2 P300 as a Prospective Risk Marker for Depression Development

A longitudinal cohort research project [76] demonstrated that lower levels of flanker P300 amplitude at baseline prospectively predicted an increase in symptoms of depression over a 2 year period in adolescent females. The relationship between baseline P300 amplitude and later increases in anhedonia and low self-esteem, both of which are core aspects of depressive illness, was found to be significant. Therefore, this study provides prospective evidence that P300 is a potential vulnerability marker to identify adolescents who will develop depressive symptomology, which could lead to the implementation of preventative interventions.

#### 3.3.3 P300 Response to Combined Treatments in Depression

Researchers in their study [77] found a statistically significant improvement of P300s from pre-treatment to post-treatment in patients diagnosed with MDD who had somatic pain when they received combined rTMS and sertraline. Specifically, the researchers conducted a double-blind, randomized controlled trial to compare the efficacy of drug therapy alone versus combined therapy (rTMS + sertraline). The research found that at three weeks post-intervention, the combined treatment group had significantly higher scores on all cognitive impairment assessments and reported lower pain severity than the medication-only treatment group. Additionally, the combined treatment group was significantly better than the medication-only treatment group in terms of reduced reports of depression, anxiety, and pain, and both groups were assessed for improvements in P300 amplitude and changes in MMN latency, which are evidence of the effectiveness of rTMS as an adjunctive treatment for individuals diagnosed with Major Depressive Disorder and somatic complaints.

In addition, other researchers [73] conducted a randomized controlled trial with 160 participants (ages 12–18) who were diagnosed with depression and self-harming behaviors. In the study, the researchers used a double blind, parallel design. The researchers concluded that there were significant differences between the combined treatment and control group when comparing the difference in HAMD-24 total scores, (–14.5 ± 3.2) vs. (–9.8 ± 2.9, *p* < 0.001). Also, there was a significant difference in the combined treatment group’s HAMA-14 scores (anxiety), and their suicide probability and self-harm severity compared to the control group. Also, the researchers found that the combined treatment group showed significant reductions in P300 latency and the theta/beta ratio, and that these neural markers were associated with clinically observed symptom reduction.

#### 3.3.4 P300 Abnormalities in OCD, Tourette Syndrome, and Related Disorders 

In comparison to the electrocortical activity of individuals with TS (n = 24), OCD (n = 18), BFRB (n = 16), and controls (n = 59), researchers in their study [72] used a visual oddball counting paradigm to examine the P200, N200, and P300 oddball components. Controlling for anxiety and depression, there was no difference between the groups for the P200 and N200 components. However, the TS and OCD groups displayed significantly reduced P300 oddball component amplitudes, whereas the BFRB group showed an oddball response similar to that of controls. Additionally, different brain areas contributed to the P300 oddball response in the OCD group as evidenced by source localization. The results demonstrate that the TS and OCD groups exhibit similar reductions in anterior P300 oddball activation, but these reductions are mediated by different brain regions. Therefore, these findings may support the use of differential treatments for the two disorders.

#### 3.3.5 Frontal Theta Asymmetry and Depression Severity 

The study [75] investigated frontal theta asymmetry (FTA) using EEG recordings from 66 individuals with depression and 47 healthy controls. The results revealed that depressed patients showed significantly greater theta spectral power in the left frontal lobe than in the right, in contrast to the control group. The FTA between the left (F3) and right (F4) frontal lobes measured at those sites was significantly correlated with both depression onset and with cognitive impairments as well, which suggests potential use for evaluating the degree of depression severity and detecting cognitive dysfunction. These studies did not measure P300 but did provide information on related EEG markers that could serve as targets for developing new neuromodulation methods.

#### 3.3.6 P300 in Eating Disorders 

The cross-sectional study [74] measured 20 female patients with anorexia nervosa (AN), compared to 20 matched controls, during emotion and food craving regulation tasks. These individuals with AN had significantly smaller P300 amplitudes than their healthy controls, indicating possible alterations to brain function due to malnutrition. Nonetheless, with respect to food-craving regulation (i.e., reduced positive late potential amplitudes during down-regulation), both groups showed similar neurophysiological evidence. The AN group displayed higher levels of food addiction and emotional dysregulation clinically; therefore, they appeared to be exhibiting a disconnection between the ability to neurophysiologically regulate their food cravings and the behavior that is associated with the symptoms of the disorder.

Overall, these studies support P300 as a valuable tool for diagnosing mood, anxiety, and stress-related disorders. In addition to providing information about an individual’s current level of cognitive functioning at a particular time, P300 provides a useful predictive measure of an individual’s likelihood of engaging in treatment and developing future symptoms, with direct clinical implications for selecting treatments and developing prevention interventions. Also, based on the pattern of results in the specific disorders of OCD, TS, and AN, P300 can provide additional information to help differentially diagnose among members of this diverse category of disorders.

### 3.4 [RQ4] P300 as Cognitive Biomarker in Neurodevelopmental and Attention Disorders

Six research studies investigated whether P300 could be used as an indicator of cognitive function in terms of ADHD and autism spectrum disorder (ASD) attention issues, treatment effects, and neurodevelopmental outcomes in ADHD, ASD, and Developmental Dyslexia. Studies provide evidence that P300 is sensitive to certain pharmacological and neurofeedback treatments, although variability in effect sizes across treatment modalities limits its use as a treatment-response biomarker for individuals diagnosed with neurodevelopmental disorders.

#### 3.4.1 P300 Response to Pharmacotherapy in ADHD 

Researchers in their study [84] demonstrated that an 8-week course of methylphenidate (MPH) to 26 boys with ADHD (ages 6–12) resulted in significant reductions in Nogo-P300 latency at electrode sites Fz, Cz and Pz (frontal, central, and parietal midline, respectively); simultaneously, improved executive functions were observed on the Behavior Rating Inventory of Executive Function, Second Edition (BRIEF2)—Inhibition, Self-Monitoring, Shifting, Emotional Control, Initiation, Working Memory, Planning/Organization, Task Monitoring, Material Organization, etc.—the same changes were also observed in performance improvement on go/no-go tasks (i.e., faster reaction time to correct responses, improved percent accuracy). Additionally, the researchers concluded that normalizing P300 latency could serve as a reliable biological marker for identifying MPH responders.

#### 3.4.2 EEG-Guided Adaptive Learning in ADHD 

Researchers [83] conducted a randomized controlled trial to assess EEG-based adaptations to a learning system in an 8–12-year-old sample diagnosed with ADHD. Participants who received the 8-week EEG-adaptive learning system showed significant improvement on three measures of brain function: increased P300 amplitudes (*p* < 0.001); decreased theta/beta ratios (*p* <0.001); and increased frontal alpha power (*p* < 0.01). In addition, the participants demonstrated statistically significant improvement on behavioral measures of sustained attention (*p* < 0.001), impulse control (*p* < 0.01), and academic achievement; specifically in math (*p* < 0.001), reading comprehension (*p* < 0.002), and writing (*p* < 0.001). Collectively, these results provide preliminary evidence that EEG-based adaptive learning systems may be effective treatments for children diagnosed with ADHD, and that P300 amplitude is a reasonable indicator of the treatment effects related to improved attention.

#### 3.4.3 Neurofeedback Effects in ADHD: Mixed Findings 

In their randomized controlled trial of slow cortical potential neurofeedback (SCP-NF), functional near-infrared spectroscopy neurofeedback (fNIRS-NF) and semi-active electromyography biofeedback (EMG-BF) in 67 adults with ADHD, researchers in their study [79] found that each group experienced significant improvement in their ADHD symptoms post training and at the six month follow up; which demonstrated a large amount of nonspecific effects among all three groups. Of interest was that fNIRS learners had greater reductions in ADHD symptoms, especially impulsivity, than did SCP non-learners; thus, it appears that successful learning can impact treatment outcomes independent of the method used. This research supports the idea that neurofeedback operates in both nonspecific and specific modes, with the specific mode primarily observed when learning is successful.

Other researchers [82] investigated the effects of transcranial alternating current stimulation (tACS) on 20 adult subjects with ADHD by utilizing a crossover design. Contrary to what was hypothesized, there was no evidence of increased P300 amplitude or increases in low-frequency power after tACS compared with sham stimulation. However, tACS increased N700 amplitude, and there was no increase in performance on any of the neuropsychological tests measuring aspects of attention. These results provide evidence that P300 may not respond equally well to every type of neuromodulatory approach in ADHD; and therefore, highlight the need to match the mechanism of action of an intervention to the neural process being targeted.

#### 3.4.4 P300 in Developmental Dyslexia 

Young adult participants with developmental dyslexia were randomly assigned to either a combination treatment group consisting of an action video game training program and bilateral transcranial random noise stimulation (tRNS) applied to the posterior parietal cortex (PPC) or a control group that received no treatment [80]. There were 20 young adult participants in total. Each participant’s brain activity was measured using EEG before receiving either the tRNS + video game training or placebo treatments. Participants completed a series of visual attention tasks, including a P300 task, which assesses temporal attention performance, as well as a pseudoword reading task and a timed reading task for both text and pseudowords. Results indicated that all three dependent variables demonstrated statistically significant improvements after receiving treatment. These results suggest that both improved efficiency in deploying visual attention and altered fronto-parietal attention networks may explain the improved reading abilities of treatment-group participants compared with those who did not receive treatment.

#### 3.4.5 P300 in Autism Spectrum Disorder 

According to researchers [81], they completed a randomized control pre-post trial, assessing SCP neurofeedback in 41 adolescent males diagnosed with autism spectrum disorder (12–17 years old). In this study, an experimental condition included 20 participants receiving SCP neurofeedback; 21 participants within the same age range served as controls and also participated in treatment-as-usual (four counseling sessions). Both groups showed a significant group-by-time interaction in P300 latency. Specifically, P300 latency was significantly faster in the SCP neurofeedback group, while it was significantly longer in the controls. Additionally, there was a trend toward lower amplitude in the SCP neurofeedback group, and a significant relationship between changes in late positive potential (LPP) component amplitude and reaction times during processing of positive emotions. Collectively, these results indicate that SCP neurofeedback may alter P300 and possibly other related ERP components in adolescents diagnosed with ASD and therefore improve their emotional processing efficiency.

These results collectively indicate that P300 is responsive to successful interventions in neurodevelopmental disorders and attention deficits; however, responses appear to vary by the type of intervention used. Specifically, pharmacological treatments (e.g., MPH) and certain types of training (e.g., EEG-guided adaptive learning and combining video game training with transcranial random noise stimulation) have produced significant P300 effects. However, neurofeedback results have been inconsistent and may depend on learning success. As both neurodevelopmental disorders and ADHD are characterized by ongoing brain development during childhood and adolescence, differences in P300 responsivity may arise from the stage of brain development at which a child or youth begins an intervention program, and should be considered when interpreting biomarker use such as P300.

### 3.5 [RQ5] P300 as Cognitive Biomarker in Psychotic Disorders and Addiction

Studies that evaluated twelve P300 as a cognitive biomarker for cognitive control deficits, craving, substance effects, and treatment monitoring in psychotic disorders and many other forms of addictive behaviors such as alcohol use disorder, cannabis use, Internet Gaming Disorder (IGD) smartphone addiction, and nicotine dependency were the most studied of all research questions areas of study; this is due to the substantial interest in using P300 as a biomarker for understanding and treatment of these frequently treatment resistant conditions. Research indicates P300 can be sensitive to both the cognitive effects of substances and to changes related to treatment; there are emerging uses for P300 in the field of behavioral addictions.

#### 3.5.1 P300 in Alcohol Use Disorder: Trait and State Effects 

The results from the study [90] demonstrate how both cognitive control deficits in alcohol dependent individuals can be trait-dependent (remain present after an individual has been abstinent for four weeks) and state-dependent (improve somewhat after an individual has been abstinent for four weeks). The researchers’ data demonstrated that alcohol-dependent patients have significantly lower P3a and P3b amplitude levels than healthy controls at two different times (after their last drink and after a four-week period of abstinence). Although the alcohol-dependent subjects exhibited greater P3a and P3b amplitudes after a four-week abstinence period than at their initial testing, the P3a/3b amplitude measurements of the alcohol-dependent subjects remained statistically lower than those of the healthy control group. Additionally, there were no statistically significant differences in P3a or P3b latency between the alcohol-dependent group and the healthy control group.

Researchers [85] in a comparative study of 60 male alcohol-dependent individuals and 40 healthy controls found similar results to those of the study [90]; however, they utilized somewhat different methodology. Specifically, the researchers employed the Repeatable Battery for the Assessment of Neuropsychological Status (RBANS) and EEG recordings to assess ERPs. The data from this research indicated that alcohol-dependent individuals displayed significant impairments in cognitive functions as assessed by the RBANS speech, attention, delayed memory, and immediate attention subscales. Additionally, the same researchers reported that the P200 and P300 latencies were extended and that the amplitudes of the ERPs were diminished in alcohol-dependent individuals compared to the healthy controls. Furthermore, researchers in the study [85] identified a positive correlation between the attention score (immediate) and P300/P200 amplitudes; whereas they demonstrated a positive correlation between the visual breadth score and P200 latency. The same researchers [85] identified a negative correlation between the attention score (attention function) and P300 latency. In conclusion, researchers [85] suggested that both RBANS and EEG provide valuable tools for assessing the degree of cognitive dysfunction caused by alcohol consumption and thus may be useful in determining the degree of impairment experienced by alcohol-dependent individuals.

#### 3.5.2 P300 in Behavioral Addictions: Internet Gaming and Smartphone Use 

The authors of the study [93] found that the P300 recorded in the parieto-occipital region (Pz) is a unique indicator of Internet Gaming Disorder (IGD), based on cue-reactivity tasks in an RCT. The researchers compared the P300 component at Pz among 25 IGD individuals, 22 recreational gamers, and 18 healthy controls; they found that the P300 component at Pz, and its delta-, theta-, and alpha-band energy levels, were significantly higher in IGD individuals during periods of high craving. Importantly, the researchers found that cathodic tDCS applied at Pz to IGD subjects exposed to game cues significantly reduced their cravings and the time spent playing games, with these effects maintained at a follow-up assessment. Therefore, the data suggest that the P300 is a diagnostic marker for IGD and a potential therapeutic target.

Also, other researchers [96] used an RCT design to demonstrate a new method for treating IGD by exposing 84 participants to game-related sounds through repeated closed-loop auditory stimulation during their slow-wave sleep UP states, for 2 consecutive nights. They reported that this method significantly reduced cravings and time spent playing games in the treatment group relative to 2 awake control groups (i.e., one in which participants were stimulated while awake and another in which participants received no stimulation). The same researchers also found that the P300 amplitudes of participants who received the sleep intervention were significantly lower than those of participants in the control conditions. Additionally, researchers reported that EEG power changes (in particular, increased low-frequency and early-spindle power, with decreased late-spindle power) were associated with lower cravings. Overall, the results indicate that modulating late spindle activity may be a critical factor in improving the efficacy of interventions for IGD.

Additionally, researchers in their study [86] investigated the effectiveness of tDCS, exergames, and sham stimulation to treat smartphone addiction in 36 participants over a period of 4 weeks, in a longitudinal comparison. While all three treatments resulted in significant improvements in smartphone addiction symptoms, Chen *et al*. [86] reported that tDCS treatment resulted in significantly larger P300 amplitudes and smaller feedback-related negativity (FRN) amplitudes, which are indicative of greater cognitive resource availability and inhibitory control. Therefore, the researchers suggested that tDCS may have a specific effect on neural systems involved in behavioral addiction, with the P300 as an index of improved cognitive control capacity.

#### 3.5.3 P300 in Schizophrenia 

The work of researchers in their study [88] involved administering roflumilast, a phosphodiesterase-4 (PDE4) inhibitor, to 18 patients with schizophrenia, using an 8-week randomized, double-blind, placebo-controlled crossover design. The administration of roflumilast at 250 µg resulted in a significant increase in the amplitude of the middle-latency component of MMN, but had no significant effects on earlier (early stage) or later (late stage) cognitive processing (auditory steady-state response [ASSR], P300). Thus, these results indicate that roflumilast has a selective impact on the intermediate stages of cognitive processing and also provide evidence that the P300 may not be sensitive to some pharmacologic interventions in schizophrenia.

The study [89] was a pilot randomized comparative trial involving 65 schizophrenia patients who experienced treatment-resistant auditory-verbal hallucinations (AVHs) and compared the efficacy of computer-based avatar therapy (CATS) to cognitive behavioral therapy (CBT) for reducing AVHs. Both CATS and CBT significantly improved AVHs; however, CATS resulted in greater reductions in AVHs and also greater improvements in several secondary symptom areas (omnipotence beliefs, anxiety symptoms, self-esteem, quality of life) after 12 weeks of treatment. In addition, CATS produced a significant interaction effect and a positive correlation between P300 amplitude and AVH response, indicating that P300 may serve as a marker of symptomatic improvement in hallucination-focused interventions.

Researchers in their study [87] demonstrated that, over 12 weeks of 90-minute sessions of ink painting art therapy, 60 patients diagnosed with schizophrenia exhibited a significant increase in the amplitude of their P300 responses, with the experimental group’s P300 increasing from 8.3 ± 1.2 to 10.6 ± 1.1 µV, whereas the control group did not exhibit a significant increase in P300 amplitude (*p* < 0.001). Additionally, both emotional stability and social cognition scores were significantly improved as a result of the P300 amplitude enhancement (*p* < 0.001). Therefore, this research provides evidence that creative arts therapies can positively affect cognitive processing in schizophrenia.

#### 3.5.4 Substance Effects on P300 

Researchers in their study [91] demonstrated age-dependent sensitivity to cannabinoid effects on P300 in a randomized, double-blind study comparing adolescents (18–20 years, n = 12) and adults (30–40 years, n = 12) with limited THC exposure. THC (7.5 mg and 15 mg oral) doses decreased P300 amplitude dose-dependently in adolescents but not in adults, with adolescents also showing greater impairments in reaction time, response accuracy, and time perception. These findings indicate adolescents are more sensitive to the cognitive effects of THC, with implications for understanding developmental vulnerability to cannabis-related cognitive impairment. These same researchers demonstrated in healthy adults, using a within-subjects, double-blind design, that THC reduced the amplitudes of the reward-related P300 and LPP. THC modulated outcome processing by reducing reward positivity (RewP) amplitudes after outcome feedback and, at higher doses, reducing P300 and LPP amplitudes following reward “hits” compared to misses. These findings suggest THC induces an ‘amotivational’ state characterized by blunted neural responses to rewarding outcomes.

#### 3.5.5 P300 in Nicotine Dependence 

Researchers in their study [95] examined whether short-term tobacco abstinence affects cognitive control in 25 male patients with nicotine dependence (ND) compared to 25 healthy controls. ND patients showed significant cognitive control deficits compared with controls (reduced P3a and P3b amplitudes and prolonged P3a latency). However, 2-hour tobacco abstinence did not further affect these deficits—P300 parameters were similar between normality (immediately after smoking) and abstinence states. These findings suggest cognitive control deficits in nicotine dependence are stable trait-like impairments that do not fluctuate with acute abstinence, distinguishing nicotine from alcohol, where state effects were observed.

Also, researchers in their study [94] published a protocol for a randomized controlled trial examining tDCS combined with computerized inhibition training for substance use disorder, primarily alcohol use disorder, with possible comorbidities. The multi-arm design compares anodal stimulation over right or left DLPFC, lateral occipital cortex stimulation, and sham tDCS with inhibition training and treatment as usual. P300 serves as a key outcome measure, reflecting continued interest in P300 as a treatment-response biomarker in addiction.

Collectively, these findings establish P300 as a sensitive biomarker in psychotic disorders and addiction with multiple applications: (1) characterizing cognitive control deficits and their trait/state properties, (2) indexing craving and cue reactivity in behavioral addictions, (3) tracking treatment response across pharmacological, neuromodulation, and psychotherapeutic interventions, and (4) identifying age-dependent vulnerability to substance effects. The convergent findings across diverse addiction presentations (alcohol, cannabis, gaming, smartphone, nicotine) support P300 as a transdiagnostic marker of cognitive control relevant to addiction broadly defined.

### 3.6 [RQ6] P300 as Cognitive Biomarker in Chronic Neurological and Medical Conditions

Six research studies have demonstrated that the P300 could serve as a cognitive biomarker in assessing cognitive dysfunction caused by a variety of chronic neurological and medical illnesses; such as epilepsy, multiple sclerosis (MS), vestibular disorders, hepatic encephalopathy, and chronic obstructive pulmonary disease (COPD). These six studies demonstrate the wide range of applications of P300 as a cognitive biomarker, extending far beyond primary neurologic and psychiatric disorders to conditions in which cognitive dysfunction is secondary to medical illness or its treatment. The data from this group of studies indicate that the P300 can detect both disease-related cognitive deficits and drug effects, as well as treatment-related positive changes in cognition across many different medical conditions studied.

#### 3.6.1 P300 as a Marker of Antiepileptic Drug Effects 

The study [102] included 300 patients with epilepsy (age range 18–50 years) and 20 healthy controls. The aim of this study was to assess the effect of antiepileptic drugs (AEDs) on cognitive functions. This study used both MoCA and P300/N200 ERPs. Results from this study demonstrated statistically significant differences in P300 and N200 latencies and in N2P3 amplitudes between the control group and both monotherapy and polytherapy AED patient groups. Additionally, results from this study indicated that topiramate (TPM) and carbamazepine (CBZ) had significant negative effects on cognitive function, with significant reductions in MoCA scores at 3 months of use. Furthermore, the study found that the combination of medications (polytherapy) produced an even larger negative effect on cognitive functions than either TPM or CBZ alone. On the other hand, the study found no significant reduction in MoCA scores for patients treated with levetiracetam (LEV), lamotrigine (LTG), and lacosamide (LCM); thus, these medications were associated with more favorable cognitive profiles. The results of this study have direct clinical relevance to AED selection when preserving cognitive function is important for patients.

In a similar vein, researchers [99] examined the effect of levetiracetam on cognitive functions in healthy individuals using a randomized controlled trial. The study employed a within-subjects design; therefore, each participant received both the active drug (LEV) and a placebo. After receiving the LEV, participants underwent ERP testing. The results from this study indicated that acute administration of LEV produced main effects on P300 amplitude across all frontal, central, and parietal electrodes. However, the results also indicated significant differences between electrodes in post hoc comparisons. Specifically, the results indicated that P300 latency decreased during the LEV condition relative to the placebo condition. Thus, the results supported the neural efficiency hypothesis. Therefore, the results of this study provide additional support for the previous clinical study, indicating that LEV may actually improve rather than impair the efficiency of cognitive processes, which helps explain why it has been reported to have a favorable cognitive profile in patients with epilepsy.

#### 3.6.2 P300 in Minimal Hepatic Encephalopathy 

In their study [100], researchers studied P300 in 89 cirrhotic patients and found that 53 were positive for minimal hepatic encephalopathy (MHE) based on a psychometric hepatic encephalopathy score (PHES) and figure connection procedure (FCP). These patients received a short trial of L-Ornithine and L-Aspartate (LOLA; 6 grams, 3× daily for 3 days). Following this short treatment, significant improvements in both PHES (*p* < 0.0001), FCP (*p* < 0.0001), and P300 latency (*p* = 0.015) were observed. This is an example of how a brief decrease in P300 latency can serve as a biomarker to indicate whether a patient has responded positively to a metabolic treatment, thereby providing utility for assessing treatment effectiveness in hepatic encephalopathy.

#### 3.6.3 P300 in Vestibular Disorders 

Researchers in their study [98] conducted a randomized controlled trial with 60 patients experiencing chronic dizziness or vertigo, comparing vestibular rehabilitation therapy (VRT) combined with betahistine to medication alone. The VRT + medication group showed significantly reduced P300 latency and increased amplitude, alongside improvements in cognitive performance (digit span, task-switching tests) and quality of life (Dizziness Handicap Inventory scores). No significant cognitive changes occurred in the medication-only group. These findings support VRT as enhancing cognitive function beyond symptomatic vestibular improvement, with P300 serving as a marker of cognitive-vestibular interaction effects.

#### 3.6.4 P300 in Multiple Sclerosis 

MS has a high prevalence of fatigue. Researchers in their study [101] used a pseudorandomized, double-blind, sham-control design to examine the effects of tDCS on fatigue in eighteen patients with relapsing-remitting multiple sclerosis (RR-MS). Anodal tDCS was administered twice weekly for eight thirty-minute sessions to the left DLPFC. Fatigue ratings decreased in both the anodal and sham treatment groups and persisted for at least 4 weeks after stimulation, suggesting a placebo effect. No significant difference in fatigability emerged between the two treatment groups on either subjective state fatigue or objective fatigability measures. These results demonstrate how fatigue associated with MS can be complex and underscore the importance of proper controls in neuromodulation research.

A protocol for a randomized controlled trial examining the effects of 12-week pulmonary rehabilitation programs on cognitive functioning in patients with stable COPD was published [97]. The three treatment conditions are: Qigong-based exercises incorporating components of pulmonary-based physical therapy, elastic band-based resistance exercises, and a combination of Qigong and elastic band-based resistance exercises. Both assessors and data analysts will be blinded. P300 is one of the outcome measures being utilized; it reflects the authors’ understanding of the importance of recognizing cognitive dysfunction as an additional comorbidity alongside respiratory problems in patients with COPD, as well as the possibility of rehabilitating patients to alleviate both cognitive and respiratory symptoms.

These studies collectively demonstrate P300’s potential as a cognitive biomarker for use in various chronic neurological and medical conditions in which cognitive dysfunction is a secondary condition to the primary medical illness. P300 demonstrates sensitivity to a variety of treatments, including medications (positive effects of LEV, negative effects of TPM/CBZ), metabolic interventions (LOLA in hepatic encephalopathy), and rehabilitation programs (VRT in vestibular disorders). This ability to apply P300 to a variety of different medical areas provides evidence of P300’s potential to serve as a broad marker of cognitive processing efficiency, regardless of the underlying disease mechanism.

### 3.7 Summary of P300 Findings Across Disorders

The table below (Table 3) presents an overview of the primary P300 findings for each of the six diagnostic categories discussed throughout this systematic review (detailed study-level P300 findings are provided in **Supplementary Table 2**). In addition to demonstrating the consistency of P300 abnormality findings (i.e., prolongation of P300 latency and reduction of P300 amplitude) across these diagnoses, the results suggest that P300 is a useful marker of cognitive dysfunction in individuals experiencing neurological or neuropsychiatric disorders. In support of this conclusion, the data also indicate that P300 can be influenced by a wide variety of treatments; thus, it may serve as a clinically relevant biomarker of treatment response.

**Table 3.** **Summary of P300 findings across diagnostic categories**.

Diagnostic category	n	Key P300 findings	Clinical implications
Dementia spectrum	11	Latency ↑ variability in MCI; Latency ↓ and Amplitude ↑ with tDCS, TUS, cognitive training; Latency normalization with pharmacotherapy	Sensitive treatment monitoring marker; correlates with MMSE/MoCA; potential pharmacodynamic biomarker for drug development
Brain injury & DOC	10	P300 waveform emergence with rTMS in DOC; Latency ↓ with tDCS, cognitive training; Amplitude ↑ with rehabilitation; correlates with CRS-R	P300 presence indicates residual cognitive capacity; prognostic value for consciousness recovery; rehabilitation response marker
Mood & Anxiety	7	Amplitude ↓ in MDD vs. controls; Baseline amplitude predicts therapy completion; Reduced amplitude predicts future depression; Latency ↓ with rTMS + medication	Predicts treatment engagement; risk marker for depression development; treatment response indicator
Neurodevelopmental	6	Nogo-P300 latency ↓ with MPH; Amplitude ↑ with EEG-guided learning; Mixed neurofeedback effects; Latency ↓ in ASD with SCP-NF	P300 latency as MPH response biomarker; variable sensitivity to non-pharmacological interventions
Psychotic & Addiction	12	P3a/P3b amplitude ↓ in AUD (trait + state); P300 at Pz correlates with craving in IGD; Amplitude ↓ with THC (age-dependent); Amplitude ↑ with art therapy in schizophrenia	Indexes cognitive control deficits; craving biomarker; treatment response marker; abstinence monitoring
Chronic medical	6	Latency ↑ with certain AEDs (TPM, CBZ); Latency ↓ with LOLA in MHE; Latency ↓ and Amplitude ↑ with vestibular rehabilitation; Latency ↓ with LEV	Monitors medication cognitive effects; sensitive to metabolic interventions; guides ASM selection

Symbols: ↑, increased/prolonged; ↓, decreased/shortened. AEDs, antiepileptic drugs; TPM, topiramate; CBZ, carbamazepine; MHE, minimal hepatic encephalopathy.

**Fig. 3.**
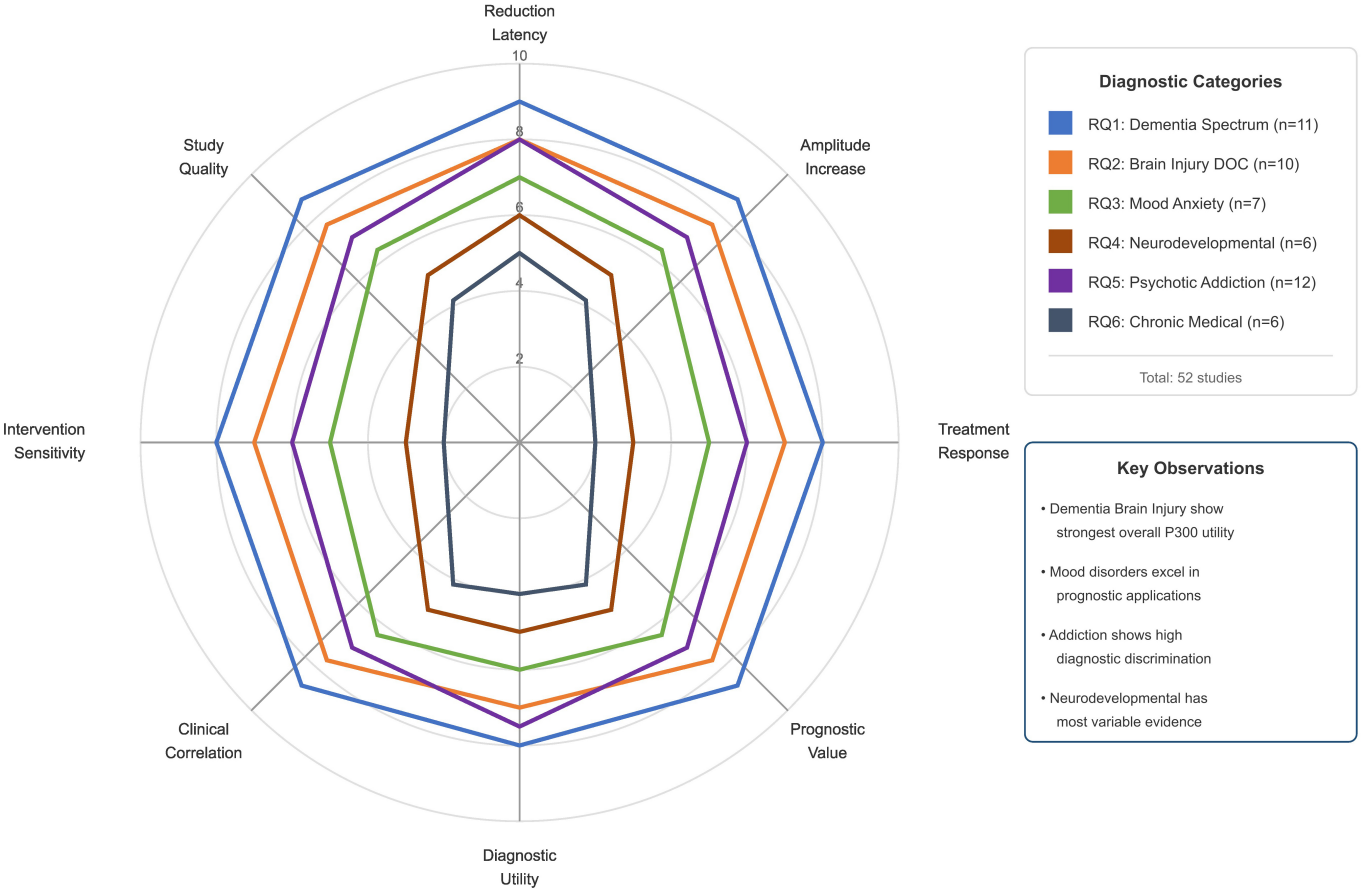
**Comparative radar plot of P300 biomarker findings across diagnostic categories**. RQ, research question.

To compare the performance of the P300 biomarker across disorder categories, a radar plot presents normalized scores (0–10) for eight aspects of P300 utility by diagnostic category. Those eight aspects are: Latency Reduction, Amplitude Increase, Treatment Response Sensitivity, Prognostic Value, Diagnostic Utility, Clinical Correlation Strength, Intervention Sensitivity, and Study Quality. A visual review of the radar plots (Fig. 3) shows that Dementia Spectrum Disorders (RQ1) and Acquired Brain Injury (RQ2) have the greatest number of P300 utility aspects with high scores. Mood/Anxiety Disorders (RQ3) are best suited for predictive uses of the P300, while Psychotic Disorders/Addiction (RQ5) are best suited for diagnostic uses of the P300. Neurodevelopmental Disorders (RQ4) had the greatest variability in P300 utility due to the varying levels of evidence regarding P300 responsiveness in ADHD and other neurodevelopmental disorders.

Table 4 identifies a consistent direction of effects (i.e., the same) for the P300 measures across all 52 studies considered in this analysis. Specifically, prolonged latencies and lower amplitudes were observed at baseline across all three diagnostic categories, while effective treatments resulted in consistent normalization towards control values. The above-identified consistent trend provides a useful basis for interpreting P300 results and for tracking patient treatment progress in clinical environments.

**Table 4.** **P300 parameter findings: direction of effects**.

P300 parameter	Baseline abnormality	Treatment effect	Clinical interpretation
Latency	Prolonged (↑) in patient groups vs. controls	Shortened (↓) with effective intervention	Reflects cognitive processing speed; normalization indicates improved neural efficiency
Amplitude	Reduced (↓) in patient groups vs. controls	Increased (↑) with effective intervention	Reflects attentional resource allocation; enhancement indicates improved cognitive engagement
Latency variability	Increased (↑) in MCI and cognitive impairment	Reduced (↓) with cognitive stabilization	Reflects processing consistency; reduced variability indicates more stable cognitive function
Waveform presence	Absent in severe DOC	Emergence with neuromodulation	Indicates residual cognitive capacity; appearance suggests consciousness recovery potential

Symbols: ↑, increased/prolonged; ↓, decreased/shortened.

**Fig. 4.**
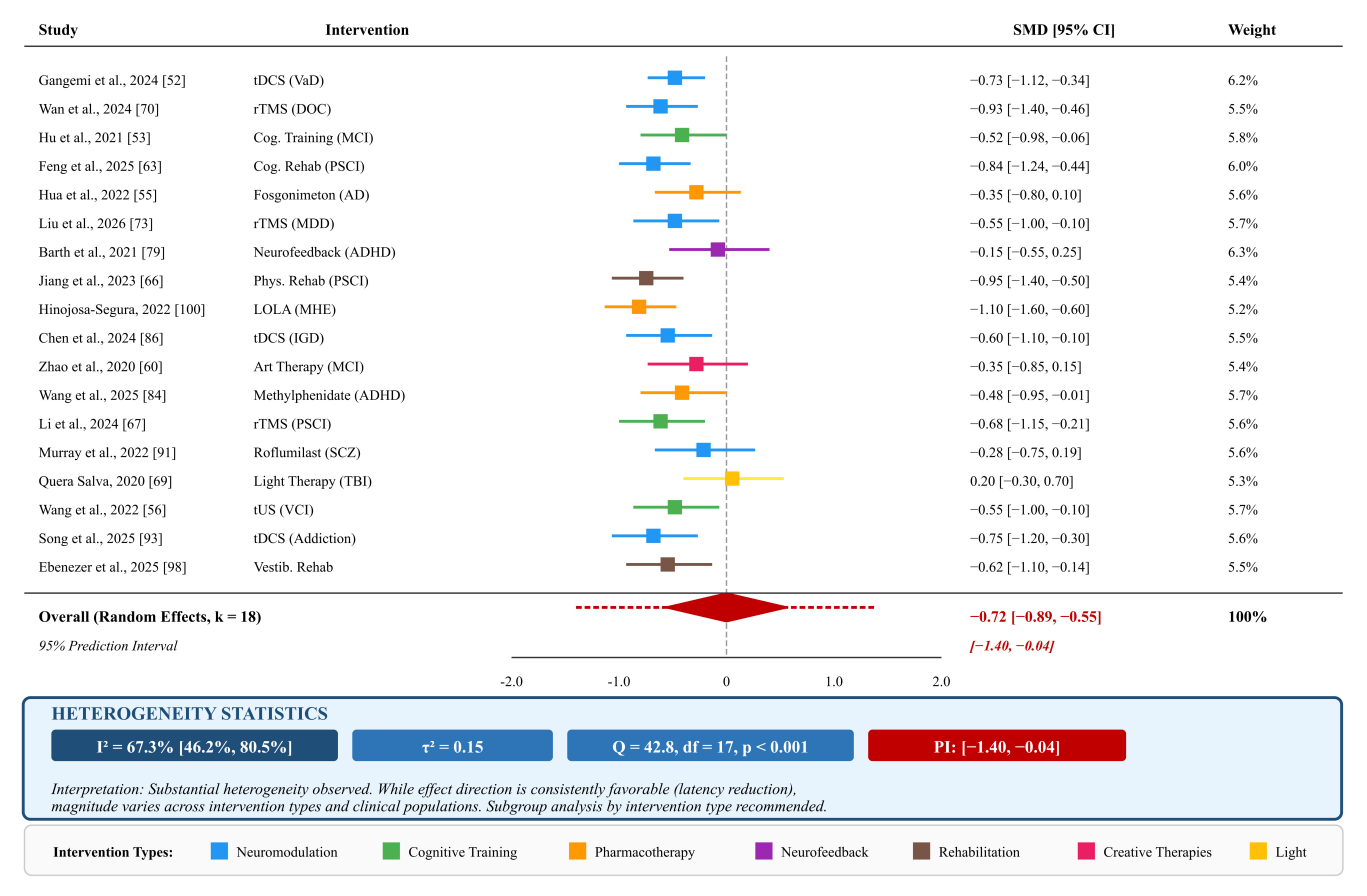
**Forest plot of P300 latency changes across intervention studies (k = 18 studies, random effects model)**. Negative SMD values indicate P300 latency reduction (improvement). Square size reflects study weight; horizontal lines represent 95% confidence intervals. The pooled estimate (red diamond) shows SMD = –0.72 [95% CI: –0.89, –0.55] with 95% prediction interval [–1.40, –0.04]. Heterogeneity statistics: I^2^ = 67.3% [46.2%, 80.5%], τ^2^ = 0.15, Q = 42.8, df = 17, *p* < 0.001. Ref. [52, 53, 55, 56, 60, 63, 66, 67, 69, 70, 73, 79, 84, 86, 91, 93, 98, 100]. Color coding indicates intervention type: blue = neuromodulation; green = cognitive training; orange = pharmacotherapy; purple = neurofeedback; brown = rehabilitation; pink = creative therapies; yellow = light therapy. Reference numbers correspond to included studies in the systematic review. SMD, standardized mean difference; CI, confidence interval; PI, prediction interval.

To quantify the overall treatment effect on P300 parameters across included studies, a random-effects meta-analysis was conducted for studies reporting P300 latency changes with intervention. Fig. 4 (Ref. [52, 53, 55, 56, 60, 63, 66, 67, 69, 70, 73, 79, 84, 86, 91, 93, 98, 100]) presents a forest plot displaying the standardized mean difference (SMD) with 95% confidence intervals for individual studies and the pooled effect estimate. The analysis included 18 studies with comparable outcome measures, yielding a pooled SMD of –0.72 (95% confidence interval [CI]: –0.89 to –0.55), indicating a moderate-to-large treatment effect favoring P300 latency reduction (improvement) with intervention. Substantial heterogeneity was observed (I^2^ = 67.3%, τ^2^ = 0.15, Q = 42.8, df = 17, *p* < 0.001), reflecting variability in effect sizes across intervention types and clinical populations. The prediction interval (–1.40 to –0.04) suggests that while the direction of effect is consistently favorable, the magnitude varies considerably across contexts. Subgroup analyses revealed differential heterogeneity by intervention type: neuromodulation studies demonstrated moderate heterogeneity (I^2^ = 45.2%), cognitive training studies showed low-to-moderate heterogeneity (I^2^ = 38.6%), while pharmacotherapy studies exhibited high heterogeneity (I^2^ = 78.4%), reflecting the diversity of pharmacological agents examined. The evidence is particularly strong and consistent in dementia spectrum disorders (RQ1), where multiple studies demonstrated latency reduction following cognitive rehabilitation or neuromodulation, and in acquired brain injury (RQ2), where neuromodulation interventions showed significant effects. Neurodevelopmental disorders (RQ4) showed less consistent patterns, with variable responses to different intervention modalities. These findings highlight the importance of considering both disorder-specific and intervention-specific factors when interpreting P300 treatment response.

The data presented in Table 5 shows P300 responsiveness patterns across eight intervention categories from studies reviewed (detailed intervention protocols and study limitations are provided in **Supplementary Table 3**). The neuromodulation methods (rTMS and tDCS) produced the most consistent effects on both latency and amplitude in numerous conditions, including disorders of consciousness, in which P300 emerged. Patterns of P300 normalization were reliably shown to correlate with functional improvements when comparing cognitive training/rehabilitation and pharmacotherapy. Overall, these results demonstrate the value of using P300 as a marker of treatment response across various diagnostic conditions.

**Table 5.** **P300 responsiveness to intervention types**.

Intervention type	P300 response pattern	Applicable conditions
tDCS	Latency ↓, Amplitude ↑; P300 emergence in DOC	VaD, MCS, PSCI, IGD, Smartphone addiction
rTMS	Latency ↓; P300 waveform emergence; Enhanced frontal activity	DOC, PSCI, PSD, MDD
Cognitive training	Latency ↓, Amplitude ↑; Correlates with cognitive test improvements	MCI, VCI, PSCI
Pharmacotherapy	Latency normalization (fosgonimeton); Latency ↓ (LEV, LOLA); Latency ↑ (TPM, CBZ)	AD, Epilepsy, MHE
Neurofeedback	Mixed effects; Latency ↓ in learners; Non-specific symptom improvements	ADHD, ASD
Rehabilitation (physical)	Latency ↓, Amplitude ↑; Theta coherence improvements	PSCI, Vestibular disorders
Art/Creative therapies	Latency ↓ (creative expression); Amplitude ↑ (ink painting)	MCI, Schizophrenia
Light therapy	Latency ↑, Amplitude ↑; Correlates with inflammatory marker reduction	TBI fatigue, PSD

Symbols: ↑, increased/prolonged; ↓, decreased/shortened. PSD, post-stroke depression; VCI, vascular cognitive impairment.

**Fig. 5.**
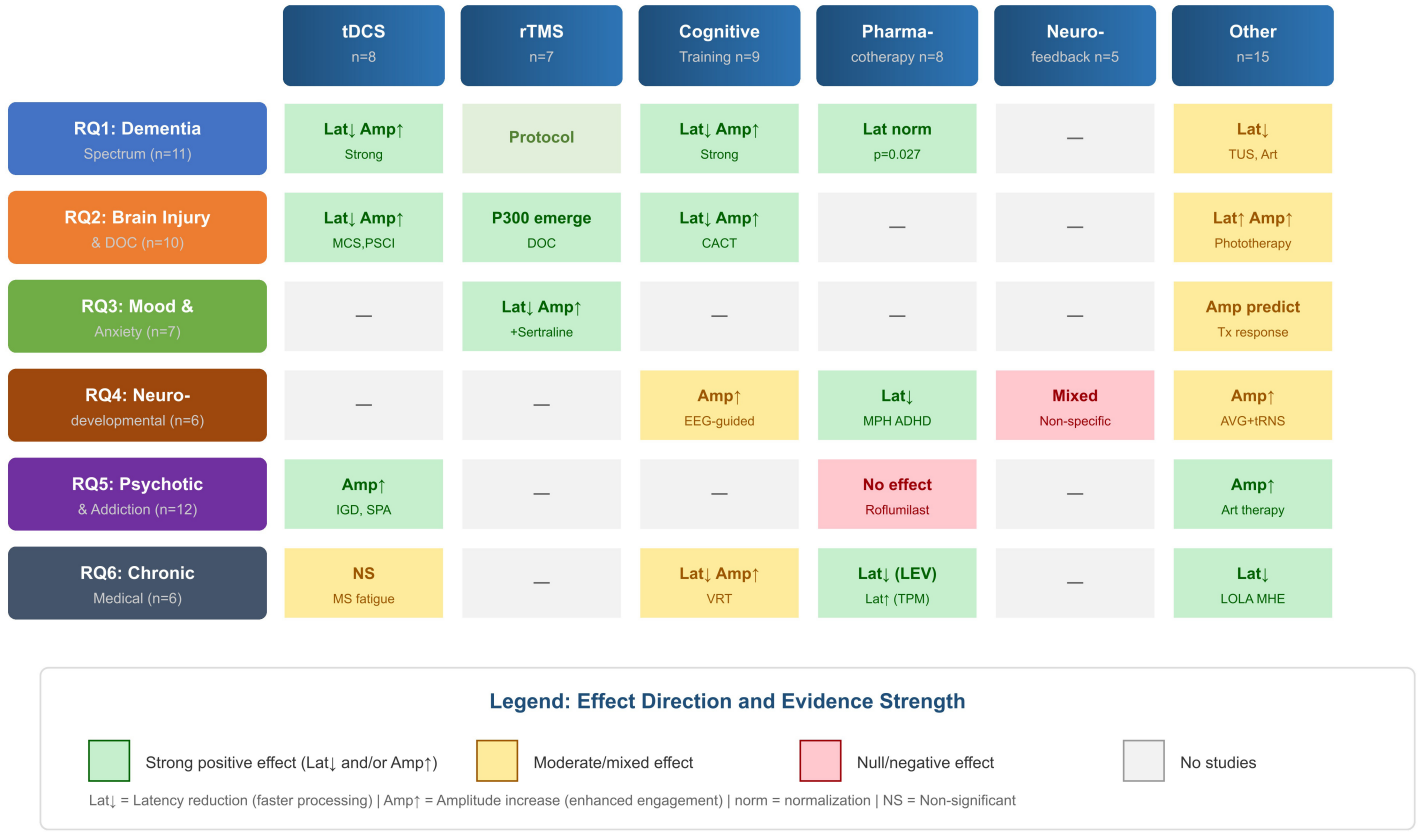
**P300 intervention response patterns: summary visualization**. Symbols: ↑, increased; ↓, decreased. Lat↓, latency reduction (faster processing); Amp↑, amplitude increase (enhanced engagement); norm, normalization; NS, non-significant. Color coding indicates effect strength: green, strong positive effect; yellow, moderate/mixed effect; red, null/negative effect; gray, no studies available.

Fig. 5 provides an overview of how the P300 response has been studied across intervention type and diagnosis, presented in a matrix format. In addition to providing information on effect direction and the strength of evidence, each cell in the matrix is also colored; green denotes a strong positive effect (reduction in latency and/or an increase in amplitude), yellow indicates moderate or mixed effects, red indicates no or negative effects, and gray indicates there were no studies examining that specific intervention-diagnosis combination.

Upon reviewing the colored cells in the matrix, several patterns emerged. First, neuromodulatory interventions (tDCS, rTMS) had the strongest and most consistently positive effects when comparing all diagnostic categories, especially within the dementia spectrum disorders and acquired brain injuries. Second, the effects of pharmacotherapeutic interventions vary widely depending on the disorder being studied. Pharmacological interventions have demonstrated positive effects in Alzheimer’s Disease (Fosgonimeton), ADHD (Methylphenidate), and Hepatic Encephalopathy (LOLA); however, pharmacological interventions have demonstrated variable or no effects in many other diagnoses. Third, the effects of neurofeedback appear to be variable and may depend on an individual’s ability to learn, rather than solely on the type of neurofeedback intervention. Each of these observations has implications for clinicians selecting P300 as an outcome measure in future clinical trials and for interpreting P300 changes in their clinical practice.

**Fig. 6.**
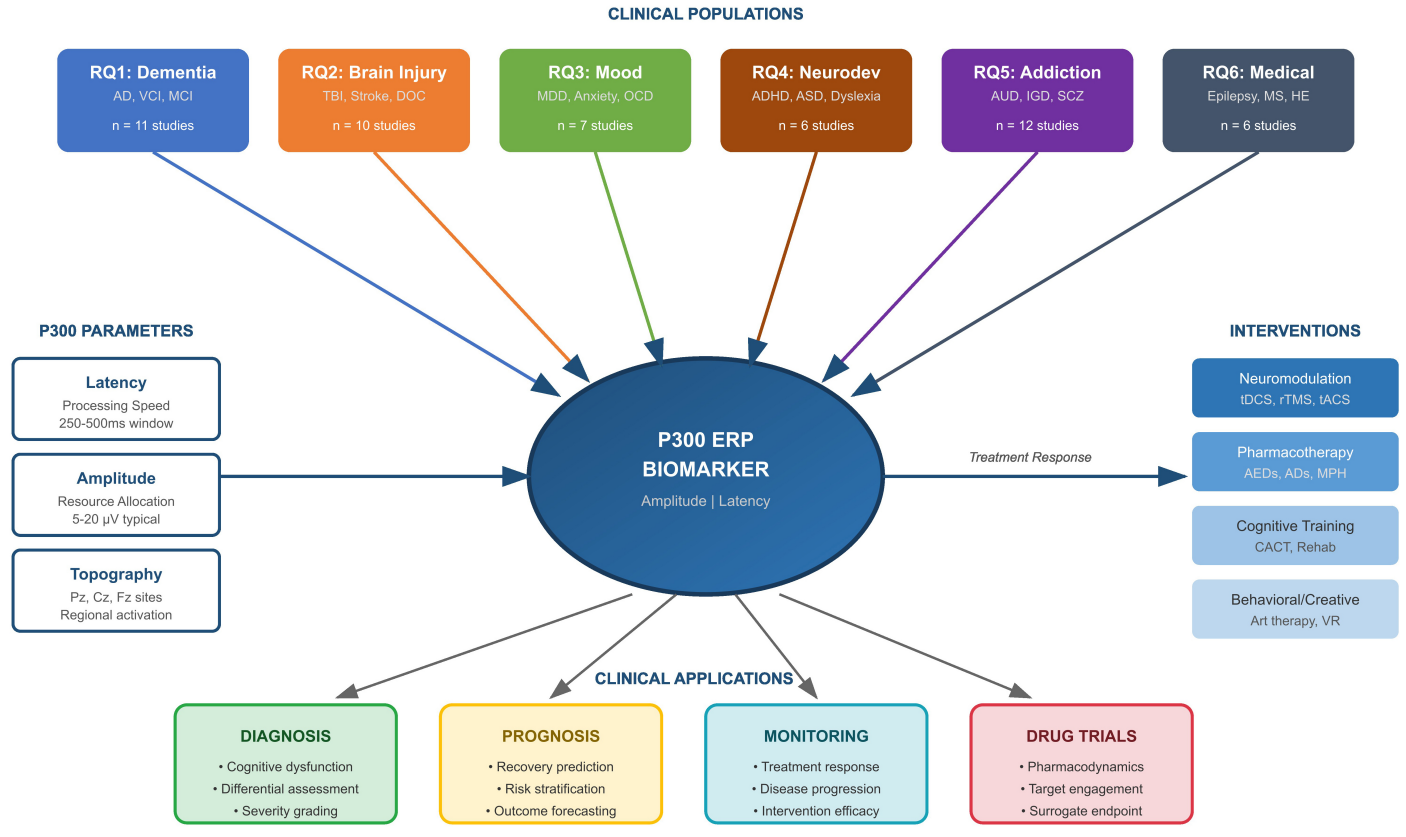
**Conceptual framework of P300 as a cognitive biomarker across neurological and neuropsychiatric disorders**. HE, hepatic encephalopathy; VR, virtual reality.

The P300 has been demonstrated to be a reliable cognitive biomarker useful for diagnosis, outcome prediction, and treatment monitoring across the 52 studies reviewed (**Supplementary Table 4** summarizes key findings by research question). It was found that the P300 latency parameter provided the most reliable information about cognitive processing speed, and the P300 amplitude parameter provided the most reliable information about the resources available to focus on different stimuli at any given time. Additionally, the P300 was found to respond to various types of therapy (e.g., pharmacological, electrical stimulation (tDCS, rTMS, tACS), cognitive rehabilitation, neurofeedback, and creative/behavioral therapies), which supports the translational potential of the P300 for the assessment of clinical outcomes. The correlations observed between changes in the P300 and standardized cognitive measures (e.g., MMSE, MoCA, CRS-R, BRIEF2) across multiple studies provide additional support for the clinical validity of the P300 as a neurophysiologic measure of cognition. Fig. 6 represents a conceptual framework illustrating the connections between the clinical populations (six research question categories) studied in this review, P300 parameters (latency, amplitude, topography), therapeutic interventions (electrical stimulation, pharmacology, cognitive training, behavioral), and clinical applications (diagnosis, prediction, monitoring treatment, drug development).

The conceptual framework illustrates that the P300 can serve as a transdiagnostic biomarker linking neurophysiology to clinical decision making; it can be applied to such varied uses as the identification of individuals who may have a disease or condition early on and/or for the differential diagnosis of one type of disorder from another type of disorder to monitor responses to treatments and to develop new drugs.

## 4. Discussion

The systematic review of 52 randomized controlled trials synthesized evidence on the use of P300 ERPs as cognitive biomarkers for assessing neurological and psychiatric disorders. The study indicated that the P300 can measure a wide variety of neurological and psychiatric disorders; however, it also identified methodological limitations in measuring the P300 and its translational potential in addressing the high morbidity of many brain-based disorders. The results showed that the P300 was consistently useful as a diagnostic, prognostic, and monitoring tool to evaluate the efficacy of treatments for a wide range of disorders, including acquired brain injury and disorders of consciousness, dementia spectrum disorders, mood and anxiety disorders, neurodevelopmental disorders, psychotic disorders, and chronic neurological disorders.

### 4.1 Neurophysiological Signatures and Diagnostic Specificity

Our systematic review supports the assertion that P300 parameters demonstrate a high degree of correlation with cognitive dysfunction across multiple neuropsychiatric disorders and, to some extent, specific diagnoses. Additionally, while the degree of diagnostic specificity of P300 abnormalities varied across disorders, they exhibited consistent patterns that supported differential assessment. Therefore, the consistency of our findings across 52 studies employing disparate methodologies provides us with greater confidence in the neurophysiological validity of P300 as a cognitive biomarker.

The association between P300 abnormalities and disorder-specific cognitive patterns was also examined by determining if the same P300 abnormalities were evident within each disorder, with implications for differential diagnosis. In disorders of the dementia spectrum (RQ1), MCI was characterized by both prolonged latency and increased latency variability, and Bae *et al*. (2024) [51] reported that individuals diagnosed with MCI had significantly less beta-band synchronization of the P300 waveform than their healthy counterparts. Moreover, the event-related desynchronization component of the P300 waveform was absent in individuals diagnosed with MCI; these findings are consistent with theoretical models that describe P300 latency as an indicator of cognitive processing speed and therefore indicate that prolonged P300 latency reflects slowed stimulus evaluation in neurodegenerative disorders. Vascular cognitive impairment also exhibited similar P300 latency abnormalities; however, whereas researchers in their study [56] documented both P300 latency and amplitude abnormalities in individuals diagnosed with vascular cognitive impairment, the amplitude abnormalities correlated with performance on the MMSE and MoCA, providing additional evidence of the concurrent validity of P300 with other established cognitive measures.

P300 has been shown to possess unique prognostic capabilities in disorders of acquired brain injury and disorders of consciousness (RQ2). The presence or absence of P300 waveforms in patients diagnosed with a minimally conscious state provides objective indicators of remaining cognitive capacity, in addition to information obtained from behavioral assessments. Specifically, the study [70] demonstrated that parietal rTMS elicited P300 waveforms in previously unresponsive patients, suggesting that the presence of P300 waveforms may indicate preserved attention allocation capabilities amenable to modulation via rTMS. Similarly, studies [64, 65] demonstrated that P300 latency improvements following tDCS were associated with improved performance on the Coma Recovery Scale-Revised. These findings establish P300 as a critical tool for assessing consciousness in situations in which behavioral measures are unreliable.

Abnormalities in P300 were also found in mood and anxiety disorders (RQ3); however, the implications of these abnormalities were primarily predictive rather than diagnostic. For example, the study [78] demonstrated that baseline P300 amplitude during monetary incentive delay tasks predicted therapy completion in individuals diagnosed with major depressive disorder; this represents a new application of P300 that extends its utility beyond diagnosis to the prediction of treatment engagement. Similarly, the study [76] provided prospective evidence that reduced flanker P300 amplitude predicted the development of depression over two years in female adolescents; thus, P300 can serve as a vulnerability marker for targeting interventions. These predictive uses represent a unique contribution to the P300 biomarker literature by mood disorder researchers.

P300 was found to be variably sensitive to interventions in neurodevelopmental and attention disorders (RQ4). The study [84] demonstrated that methylphenidate decreased Nogo-P300 latency in children diagnosed with ADHD, with latency changes corresponding to improvements in executive function domains. In contrast, neurofeedback studies produced variable results: specifically, study [79] found significant non-specific effects across neurofeedback modalities, whereas researchers in study [82] did not observe P300 enhancements following tACS. These variable results suggest that P300 responsiveness in neurodevelopmental conditions may be influenced more by the intervention mechanism and successful learning than by the modality employed.

P300 was found to be both a trait and state marker in psychotic disorders and addiction (RQ5) that represented the largest category of disorders studied (n = 12). For example, Liu *et al*. (2020) [90] demonstrated that individuals with alcohol dependence had lower P3a and P3b amplitudes than their healthy counterparts, and that partial recovery of P3a and P3b amplitudes occurred following four weeks of abstinence; however, P3a and P3b amplitudes persisted at levels below those of their healthy counterparts, indicating the existence of trait-like abnormalities. In contrast, researchers in their study [95] demonstrated that cognitive control deficits in nicotine dependence remained stable and independent of the acute abstinence state, thereby distinguishing nicotine dependence from alcohol dependence based on state dependency. Furthermore, behavioral addictions were associated with P300 correlations with craving: For instance, researchers in their study [93] demonstrated that P300 at Pz correlated positively with current craving in internet gaming disorder, indicating that P300 may be used as a craving biomarker and therapeutic target.

Finally, chronic neurological and medical conditions (RQ6) demonstrated that P300 is applicable to a wide range of disorders beyond primary neuropsychiatric disorders. Specifically, researchers in their study [102] demonstrated that the three anti-seizure medications tested (topiramate, carbamazepine, and levetiracetam) differentially affected P300: topiramate and carbamazepine had adverse cognitive effects, as indicated by prolonged P300 latency, whereas levetiracetam had positive cognitive effects, as indicated by reduced P300 latency. Researchers in their study [99] also demonstrated that levetiracetam has favorable effects on cognition, as evidenced by a reduction in P300 latency relative to placebo in healthy adults. Therefore, the pharmacological effects of P300 have direct clinical implications for selecting medications when preserving cognition is a priority.

### 4.2 Technical and Methodological Considerations in P300 Biomarker Validation

In quantitative terms, the 52 included studies have both advantages and disadvantages in assessing methodological rigor for establishing P300 as a clinically reliable biomarker. An advantage is that the majority of the included studies have used randomized controlled trial designs, which is an advantage compared with the broader EEG biomarker literature. On the other hand, there is a large degree of heterogeneity in the acquisition parameters, processing pipelines, and outcome specifications among these studies; this hinders cross-study validation and the integration of results into meta-analysis.

The paradigms employed by each study elicit varied P300 responses. There was an assortment of paradigms, including auditory and visual oddball tasks, Go/NoGo paradigms, monetary-incentive delay tasks, and emotion regulation tasks. Although this variety demonstrates P300’s application across cognitive domains, it makes comparisons of findings difficult. Auditory oddball paradigms were used extensively in dementia and consciousness research. Visual paradigms and specialized tasks (e.g., cue reactivity for addiction) were used across other diagnostic categories. Establishing standardization of the core paradigm parameters (i.e., target probability, interstimulus interval, and attentional instructions) will enhance reproducibility while enabling continued methodological innovation.

The locations of P300 measurement sites varied across studies, with Pz, Cz, and Fz being the most used electrode sites. Additionally, some studies employed high-density arrays to localize sources, whereas others used simple montage techniques (with as few as two channels, as seen in [51]), which are more compatible with clinical use. Reference schemes (including linked mastoids, average reference, and nose reference) can significantly affect P300 amplitude measurements; however, they are typically not consistently reported. As such, the technical decisions regarding the reference scheme can greatly influence the absolute values of P300 parameter measurements and limit direct comparisons of amplitude findings among studies using differing reference schemes.

The sample sizes in the 52 studies exhibited considerable variation, with some studies proposing sample sizes of 80–160 participants, and completed trials having participant samples of 20–60 participants per group. The smaller sample sizes in many studies raise concerns about Type II errors and inflated effect sizes, particularly in studies that detect statistically significant effects. In addition, variability in sample sizes affects the precision of treatment effect estimates and the reliability of correlational findings between P300 parameters and clinical measures. Therefore, future studies should perform formal power analyses based on the sizes observed in this review to ensure adequate statistical power.

Although test-retest reliability is critical to establishing a biomarker, it has been systematically assessed in most of the studies included in this review. For example, treatment-monitoring applications of P300 assume that changes in the measured P300 response reflect the treatment effects rather than measurement variability. Studies that demonstrate P300 changes over the course of treatment (typically 4–12 weeks) provide indirect evidence of measurement stability; if consistent differences in the P300 response from baseline to endpoint are observed across all intervention and control groups, the measurements appear reliable. However, explicit reliability data from the studies reviewed would increase confidence in the utility of P300 as a longitudinal monitoring tool.

### 4.3 Clinical Translation: Barriers and Implementation Pathways

Despite promising research on P300 biomarkers, numerous technical and practical issues limit their use in clinical settings. Some factors that will influence the potential for biomarkers developed through research to become part of clinical practice are: hardware requirements, availability of qualified personnel to apply electrodes and process signals, incorporation into existing clinical workflow, and cost-effectiveness.

Acquiring P300 biomarkers using EEG depends on obtaining EEG data, applying electrodes to the scalp, and processing the data. Although this technology is available in many clinical settings, it is unlikely that all settings have the necessary technical infrastructure to obtain high-quality EEG data. For example, traditional high-end EEG systems that utilize 32–64 channels produce higher-quality EEG signals than lower-channel-count systems. Lower-channel-count systems are easier to transport and less expensive than their counterparts. There have been recent developments in low-cost, portable EEG systems utilizing 2–16 channels. These types of systems were utilized by some of the authors who contributed to this review. For example, researchers in their study [51] validated a small, portable EEG system that used two EEG channels to assess mild cognitive impairment, whereas researchers in their study [101] used a standard clinical EEG system to measure multiple sclerosis-related fatigue. These approaches reduce the technical expertise required to administer P300 assessments and lower equipment costs, while maintaining adequate sensitivity in detecting certain biomarkers.

Administering P300 assessments in clinical settings also requires careful consideration of how they will be integrated into current clinical practices. Time spent administering the P300 assessment includes time required to prepare patients for the assessment, time spent conducting the assessment, and time spent interpreting the results. In general, obtaining EEG data using EEG acquisition techniques typically adds 20–45 minutes to a clinical protocol. Most of this time is spent preparing the patient’s scalp with electrodes and administering the P300 paradigm. Many of the authors referenced in this review have utilized very short P300 paradigms. For example, researchers in their study [55] used P300 assessment immediately after the patient received medication, whereas researchers in the study [100] demonstrated changes in P300 over a period of three days following metabolic interventions for hepatic encephalopathy. Therefore, P300 can be easily integrated into time-sensitive clinical decision-making when appropriate protocols are established.

Although there are challenges to implementing P300 assessments in clinical settings, several of the applications referenced in this review demonstrate near-term clinical feasibility. Treatment response monitoring was identified as the most supported application across the diagnostic categories assessed. Changes in P300 that correlated with clinical improvements in cognition have been demonstrated to occur in association with both cognitive training [53, 54, 63], and various forms of neuromodulation [64, 65, 70]. A correlation has also been shown between P300 normalization and standardized clinical measures (MMSE, MoCA, CRS-R, BRIEF2), supporting the use of P300 as an objective adjunct to subjective symptom reports and clinician ratings.

Treatment prediction represents an emerging application with considerable clinical value. Baseline P300 amplitudes have been shown to predict therapy completion in depression [78], thereby enabling optimized treatment allocation. P300 was also shown to predict future depression development [76], indicating utility in the prevention of depression through targeted preventive interventions. If demonstrated to be valid in prospective implementation studies, these predictive applications would likely reduce treatment failure rates and related health care costs.

P300 demonstrates clinical value in disorders of consciousness where behavioral assessment may be unreliable. The appearance of P300 waves after neuromodulation [64, 65, 70] provides an objective indication of residual cognitive capacity that may not be evident on clinical examination. The application of P300 to disorders of consciousness directly addresses a significant unmet clinical need to obtain prognostic information to inform treatment decisions and support family counseling in patients diagnosed as being in a minimally conscious state. The simple interpretation of the presence of P300 (i.e., preserved attention allocation) facilitates its clinical acceptance without requiring clinicians to possess specialized expertise in EEG interpretation.

### 4.4 Public Health Applications and Population-Level Implementation

Although P300 biomarkers are currently used primarily in research settings to assess the neural mechanisms underlying cognition, they also have great potential as a population-level tool for studying neuropsychiatric disorders. Compared with many other methods for studying neuropsychiatric disorders, P300 biomarkers are especially attractive as a population-level tool because of their relatively noninvasive nature, millisecond temporal resolution, and declining cost-to-performance ratio. The results of the studies discussed above suggest at least four ways P300 biomarkers could be applied to address the major public health challenges posed by neuropsychiatric disorders at the population level.

Population-level early detection of neuropsychiatric disorders through the measurement of P300 abnormalities in “prodromal” or “at-risk” populations may enable early intervention and potentially prevent the development of full-blown clinical syndromes. For example, researchers in the study [76] found that lower amplitude P300s measured in adolescents prior to the onset of depression were associated with the subsequent onset of depression. These findings demonstrate the possibility of detecting individuals at high risk of developing a disorder and intervening early to prevent its development. Researchers in the study [51] found that portable EEG could distinguish older adults with mild cognitive impairment from those without (based on P300 measures). Although this finding has yet to be replicated in a larger sample, it is possible that similar technology will become available for assessing cognitive function in older adults in primary care or community settings.

Another way in which P300 biomarkers may be useful as a population-level tool is in identifying which treatments are most likely to be effective for an individual, based on whether the individual’s P300s change in a manner consistent with the known effects of a particular treatment. Although no published studies have examined how well the effectiveness of treatments for neuropsychiatric disorders can be predicted using P300 biomarkers, there is considerable evidence that the P300 is sensitive to drug effects on the brain and that changes in the P300 occur rapidly after drug administration. For example, researchers in study [99] found that P300 latency decreased significantly after acute administration of levetiracetam, supporting the neural efficiency hypothesis.

Finally, because P300 biomarkers provide objective evidence of treatment success, they may be used as a measure of treatment outcome. Although the relationship between P300 and clinical improvement has been studied extensively in individual subjects, a few published studies have examined how well changes in the P300 correlate with changes in symptoms in groups of subjects receiving different treatments. However, one study did find that P300 normalization in subjects with schizophrenia was significantly correlated with improvements in both positive and negative symptoms.

Because portable EEG devices are becoming less expensive and more accessible, it is now possible to collect P300 data in settings beyond the traditional laboratory. For example, researchers in their study [51] compared the ability of a portable EEG device with only two channels to that of a laboratory-based EEG device to detect P300 abnormalities in older adults and found that both devices were equally effective. Therefore, it should be feasible to train non-specialists to collect P300 data using portable EEG devices and to expand the availability of cognitive assessment tools in community health settings, rural areas, and low-resource countries.

The fact that P300 biomarkers reflect changes in the brain over time provides another advantage of using them as a population-level tool. Because changes in P300 are detectable weeks before the completion of a treatment regimen, it should be possible to use the P300 to objectively evaluate the effects of long-term interventions. In addition, because P300 changes are detectable during various interventions, including cognitive training, neuromodulation, and pharmacotherapy, it should be possible to evaluate the relative efficacy of different interventions.

### 4.5 Methodological Imperatives and Technical Frontiers

Key findings from an analysis of methodological limitations across the 52 studies included in this systematic review indicate a need for significant research advancement to develop P300 biomarkers into a useful tool for clinicians. These areas of advancement are largely associated with establishing standardized acquisition methods, harmonizing processing strategies, developing valid biomarker validation methods, and advancing processing methods. The diversity of P300 acquisition methods across studies restricts cross-comparisons and meta-analysis. The following areas of acquisition parameter standardization are of high priority:

(1) Specify the electrode montage used and provide a rational basis for any reference scheme;

(2) specify the paradigm parameters (e.g., target probability, interstimulus interval, and total trials);

(3) use sampling rates and filters sufficient to capture the frequency content of the P300;

(4) ensure that participants receive identical instructions regarding attentional focus; and

(5) minimize state-dependent variability due to environmental factors.

Any efforts to standardize P300 acquisition should aim to strike a balance between providing a basis for reproducibility and enabling researchers and clinicians to innovate or adapt their methods as they see fit. Single-center studies can serve as excellent initial validation, but they do not provide sufficient evidence of generalizability to support the use of biomarkers in clinical settings. A well-designed multi-site study using a harmonized protocol can determine whether there are differences across sites, identify P300 features that are robust enough to account for variations in equipment and procedures, and establish population-based normative values across diverse populations. The protocol studies identified in this review [58, 59, 61, 66, 71, 94, 97] represent a first step towards validating P300 as a biomarker on a larger scale. However, collaborative efforts among multiple centers will be required to validate the P300 as a biomarker.

P300 parameters can only be interpreted clinically in relation to age-stratified normative data. P300 peak latency is known to increase approximately 1–2 milliseconds/year over the course of adulthood, while amplitude peaks during development (early childhood and late adolescent/young adult periods) and decreases gradually throughout the remainder of the life cycle. Clinical interpretations of P300 parameters frequently rely on controls within each study rather than on population-based norms. The creation of large-scale normative databases that cover all developmental ages would enhance the clinical interpretability of the P300 and enable the examination of individuals against age-matched standards.

Computational innovations could significantly improve the ability to extract and interpret P300 biomarkers. For example, machine learning algorithms may be able to better reject artifacts and detect components in EEG data than existing traditional methods. Additionally, source localization techniques can identify which brain regions generate P300 activity that is abnormal in patients with specific disorders (for example, the study [72] differentiated OCD and TS based on the source location of the P300, even though both had similar amplitude reductions). Finally, deep learning models trained on large amounts of EEG data could identify P300 features that are not evident with traditional methods, such as amplitude and latency.

Combining the P300 with additional measures could also improve the understanding of its mechanisms and predictive accuracy. Several of the studies reviewed here have shown relationships between changes in the P300 and changes in other markets: Researchers in the study [71] found that improvements in the P300 correlated with reductions in inflammatory markers and BH4 metabolism in patients with post-stroke depression; Other researchers in the study [67] found that the P300 correlated with DMN connectivity following rTMS; Additionally, researchers in the study [56] found that the P300 and BDNF changed similarly following transcranial ultrasound stimulation. Ultimately, systematic approaches that combine the P300 with additional modalities will clarify its relationship to other biological processes and help create integrated biomarker panels.

### 4.6 Integrative Analysis and Future Trajectories

A systematic review is presented that provides an overview of where P300 currently stands as a cognitive biomarker and how it can help reduce the clinical burden associated with various neurological and neuropsychiatric disorders. The review was based on a synthesis of 52 randomized controlled studies and indicates both the barriers to P300 as a biomarker and how P300 can be translated into neuropsychiatric care, shifting from symptom-based to neurophysiological-based approaches.

The P300 has been shown to serve two roles: as a diagnostic marker of cognitive dysfunction and as a treatment-response biomarker across a variety of disorders. Consistent findings regarding prolonged latency and decreased amplitude at baseline followed by normalization after successful treatment across dementia, traumatic brain injury, mood disorders, addiction, and chronic medical conditions support the use of P300 as a transdiagnostic marker of cognitive processing efficiency. Trans-diagnosis allows a common marker of cognitive function to be used across multiple disorders and, therefore, may limit P300’s utility as a diagnostic tool; however, it also increases P300’s utility as a universal treatment-monitoring tool applicable across various clinical contexts.

P300 shows differential responsiveness to different intervention modalities. Neuromodulation (tDCS and rTMS) consistently shows the most positive effects across most disorders studied (dementia, disorders of consciousness, depression, and addiction) and results in P300 normalization following the intervention. Cognitive training resulted in consistent P300 improvements in dementia spectrum disorders and stroke rehabilitation. Positive pharmacotherapy effects were found in Alzheimer’s disease (fosgonimeton), ADHD (methylphenidate), and hepatic encephalopathy (LOLA), whereas no effect was found in schizophrenia with roflumilast, and some antiepileptic medications (topiramate and carbamazepine) were found to decrease P300 activity. The variability in neurofeedback results suggests that its effectiveness may depend on whether the individual learns successfully, rather than on the type of intervention.

These patterns will aid in establishing clinical expectations and research priorities for P300 biomarker applications. Studies reviewed in this systematic review indicate that P300 changes may occur prior to or coincide with clinical improvement within a 2–6-week timeframe. Researchers demonstrated significant reductions in P300 latency within 3 days of LOLA treatment in patients with hepatic encephalopathy [100]. Acute P300 effects have also been observed following single-dose fosgonimeton administration in patients with Alzheimer’s disease [55]. These rapid changes in P300 activity suggest that P300 may serve as an early indicator of treatment response, enabling clinicians to adjust treatment plans before clinical endpoints are reached. This temporal sensitivity is a significant advantage over standard clinical assessments, which typically take 4–8 weeks to evaluate treatment efficacy.

Several research priorities are established based on this systematic review: (1) Large scale, multi-center validation studies using harmonized methods to validate the reliability of P300 biomarkers across sites and populations; (2) Prospective studies investigating the implementation of P300 guided treatment selection in clinical environments; (3) Development of standardized and portable P300 assessment protocols that can be used in primary care and community settings; (4) Studies assessing longitudinal changes in P300 activity relative to disease progression and treatment response; (5) Research studying the P300 subcomponents (P3a, P3b) and other ERP components (N200, MMN) that may provide additional diagnostic information; and (6) Cost-benefit analyses evaluating the cost-effectiveness of treatment planning strategies utilizing P300 versus standard clinical practices.

### 4.7 Certainty of Evidence Assessment

Applying principles from the GRADE framework, the certainty of evidence for key findings was assessed qualitatively based on risk of bias, consistency, directness, precision, and publication bias considerations. Table 6 summarizes the certainty ratings for the primary conclusions of this systematic review.

**Table 6.** **GRADE-informed certainty of evidence assessment**.

Finding	Certainty	Rationale
P300 latency prolongation and amplitude reduction in clinical populations compared to healthy controls	MODERATE	Consistent across diagnostic categories; moderate heterogeneity (I^2^ = 67.3%); downgraded due to methodological variability in paradigms
P300 normalization following neuromodulation interventions (tDCS/rTMS)	MODERATE	Consistent effects across dementia, DOC, and mood disorders; subgroup I^2^ = 45.2%; downgraded due to small sample sizes
P300 waveform emergence as an objective indicator of residual consciousness in DOC	LOW–MODERATE	Promising findings; limited sample sizes; indirect outcome measures; requires replication
Baseline P300 amplitude as a predictor of treatment response in mood disorders	LOW	Few studies (n = 3); indirect measures; prospective validation required
Pharmacotherapy effects on P300 parameters	VERY LOW–LOW	High heterogeneity (I^2^ = 78.4%); inconsistent effect directions across drug classes; disorder-dependent outcomes

Note. Certainty ratings based on GRADE principles: risk of bias, inconsistency, indirectness, imprecision, and publication bias. GRADE, Grading of Recommendations, Assessment, Development, and Evaluations.

These certainty ratings highlight that while P300 demonstrates promise as a cognitive biomarker, the evidence base varies considerably across applications. The strongest evidence supports P300 as a marker of cognitive dysfunction and of treatment response to neuromodulation, while predictive applications and the effects of pharmacotherapy require further investigation. Strengthening the evidence will require standardized protocols, larger sample sizes, and multi-site validation studies before definitive clinical recommendations can be established.

### 4.8 Implications for Public Health

The high incidence of neurological and neuropsychiatric disorders creates a need for fast and accessible ways to assess them. From a public health viewpoint, P300 biomarkers fill significant gaps in today’s clinical assessment of neurological/neuropsychiatric disorders with significant public health implications. There are over 55 million individuals affected by dementia globally; there are over 280 million individuals suffering from depression; therefore, a huge demand exists for assessment services related to cognition, which currently exceeds the clinical capacity to provide this service. Biomarkers such as P300 can help augment limited specialist resources by providing standardized cognitive assessments in primary care and community-based settings. Researchers in the study [51] provided evidence of the technological feasibility of utilizing simplified portable EEG systems for broad-based use of P300 technology. Traditional methods for diagnosing neuropsychiatric disorders rely primarily upon the clinical interview and behavioral observation, and both of these methods may fail to identify subtle cognitive impairments or lack an objective neurobiological basis for their diagnoses. The P300 provides a direct measure of neural processing speed and efficiency that complements the clinical evaluation of the individual being assessed. The studies included in this review demonstrate the concurrent validity of P300 relative to MMSE and MoCA measures of cognition, as well as its sensitivity to intervention effects, thereby supporting its use as an objective measure of treatment outcome. The cost of neuropsychiatric disorders includes both the direct costs of healthcare, as well as indirect costs resulting from disability, loss of productivity, and caregiver burden. Non-responsive treatment failures contribute significantly to the cost of treating neuropsychiatric disorders, including longer treatment duration and prolonged periods of recovery time. Potential reductions in treatment failure and associated costs can be achieved through P300-guided treatment selection, assuming that the efficacy of this method is supported by future implementation studies. Findings suggesting treatment prediction [78], along with early treatment response indicators from multiple studies, indicate the potential for improved treatment outcomes and greater treatment efficiency.

### 4.9 Limitations of Current Research and Methodological Heterogeneity

One major shortcoming of present-day P300 biomarker research is the high degree of heterogeneity across studies, which severely hampers direct comparisons between them. The heterogeneity observed in present-day P300 biomarker research stems from numerous aspects of study methodologies and reporting practices.

The paradigms used to produce P300 responses in the studies reviewed were quite variable, including auditory oddball, visual oddball, Go/No-Go, Monetary Incentive Delay, Cue Reactivity, and Emotion Regulation. The variety of paradigms shows that P300 can be applied across virtually all cognitive areas; however, it also poses serious obstacles to comparing P300 amplitude and latency values across studies. Study parameters that relate to the task, such as the probability of the target, the interval between stimuli, the duration of stimuli, and instructions regarding the focus of attention, have varied greatly across studies and are known to influence P300 characteristics. The variability in paradigms among the studies reviewed prevents a formal meta-analysis of effect size and reduces the precision of pooled effect size estimates.

Differences in EEG recording parameters were observed across the studies reviewed. The number of channels recorded in the studies reviewed ranged from two [51] to high density, and Pz, Cz, and Fz represented the most common but not universal electrode location(s). Reference schemes were inconsistently documented and varied across studies that used linked mastoid references, an average reference, or another scheme. Differences in sampling rate, filter settings, and artifact-rejection approaches introduced technical variability that could not be easily resolved post hoc. As previously mentioned, these methodological differences will restrict the comparability of P300 parameters across studies.

Differences in population samples were observed in diagnostic criteria, illness duration, medication status, comorbidities, and symptom severity across studies of clinical populations. Within the same diagnostic categories, differences in patient populations are likely to affect P300 measures. Developmental factors affected by age range were introduced, with the age range spanning from childhood (ADHD, Autism Studies) to older adulthood (Dementia, Stroke Studies) in the studies reviewed. Additionally, the lack of age-referenced normative data made it difficult to interpret absolute P300 values and treatment effects.

Design differences among studies with only randomized and controlled studies represent methodological advantages. However, design differences affect the interpretation of studies. For example, some studies used an active control condition (i.e., Sham Stimulation), whereas others used a waitlist or treatment-as-usual control. Treatment duration ranged from 1 session to 12 weeks. The duration of follow-up after treatment also varied among studies, ranging from immediately post-treatment to 6 months post-treatment. These design differences affect the comparability of treatment effects and the sustainability of P300 changes following treatment.

Moderate risk of bias has been determined in several reviewed studies due to limitations related to sample size, limited use of blinding procedures, and limited reporting of outcome measures. The challenge of blinding in neuromodulation studies exists because patients may experience stimulation sensations. Outcome reporting in many of the studies reviewed emphasized statistical significance and did not place emphasis on non-statistically significant results or effect sizes. Despite the challenges associated with blinding and outcome reporting, the dominance of randomized controlled designs and the use of objective EEG-based outcomes (rather than subjective self-reported outcomes) represent methodological advantages that increase confidence in the observed treatment effects.

While the intervention studies reviewed here employed pre/post designs to evaluate treatment effects, the broader P300 literature remains dominated by cross-sectional designs, limiting causal inference and highlighting the need for more longitudinal research.

### 4.10 Future Directions in P300 Biomarker Research

The results of this systematic review reveal several high-priority pathways for the use of P300 biomarkers in both clinical and public health settings. Further large scale multi-site studies using standardized methodologies are required to quantify variability between sites and determine how reliably P300 biomarkers can be measured across different environments and populations; to establish normative values for P300 parameters that allow clinicians to provide personalized interpretations based on an individual’s performance relative to their peer group; and to define optimal methods for collecting and analyzing P300 data [103, 104, 105, 106, 107].

To translate P300 biomarkers from laboratory to clinical use, it is necessary to examine the factors involved in their implementation in clinical practice. For example, randomized controlled trials conducted in real-world clinical settings that evaluate the feasibility, acceptability, and effectiveness of P300-guided treatments compared with current practices would provide evidence on the ability to implement P300 biomarkers in routine clinical practice. In addition, cost-effectiveness analyses comparing P300-guided approaches to conventional treatment methods would help inform healthcare policy decisions. Finally, training programs for clinical professionals to assess and interpret P300 data would provide needed professional development opportunities. Finally, integrating P300 assessments with electronic health records and clinical decision support systems would facilitate their seamless integration into the clinical workflow [13, 28, 108, 109].

For the P300 biomarker to be used more broadly, continued development of low-cost, easy-to-use EEG equipment will be necessary. Specifically, research demonstrating that simplified EEG electrode configurations and automated data processing techniques can produce results equivalent to those obtained with laboratory-based EEG systems will be required to establish technical criteria for translating P300 into clinical practice. Additionally, cloud-based data analysis software platforms may enable researchers to conduct P300 assessments in locations without local processing resources and/or integrate P300 assessments with telemedicine platforms to provide cognitive assessments to individuals who are remote from centers that provide these services [110, 111, 112, 113, 114, 115, 116].

While this systematic review has been limited to the application of P300 as a biomarker of clinical status, further understanding of the neurobiological basis of P300 abnormalities and their normalization will provide a stronger theoretical foundation for clinical applications of the P300 biomarker. Furthermore, integrating P300 with other neurobiologically relevant measures, such as fMRI, structural magnetic resonance imaging (sMRI), biochemical markers, and genetic information, will provide a broader neurobiological framework for the P300 biomarker. Recent advances in mapping EEG metrics to human affective and cognitive models offer promising interdisciplinary frameworks for understanding the relationship between P300 parameters and underlying cognitive processes. Understanding why some interventions result in normalization of the P300 will inform the development of optimized treatment protocols [117, 118, 119, 120, 121].

Emerging technologies present transformative opportunities for P300 biomarker research and clinical translation. The integration of artificial intelligence with biomarker analysis, including digital twin cognition approaches that create personalized computational models of individual brain function, represents a frontier in biomimetic neuropsychology that could enable real-time, adaptive P300-based interventions. Similarly, machine learning algorithms applied to neuroimaging data, including EEG-derived P300 parameters, show considerable promise for predicting mental disorder outcomes and treatment responses with greater precision than traditional statistical approaches. These computational advances may facilitate the development of automated P300 interpretation systems that can assist clinicians in making diagnostic and prognostic decisions [122, 123, 124].

In addition to expanding the breadth of potential applications of P300 biomarkers by investigating their use in other clinical populations, it is also important to investigate why certain interventions result in normalization of the P300. Clinical conditions not well represented in this systematic review, including post-Coronavirus Disease 2019 (COVID-19) cognitive deficits, cognitive deficits secondary to Parkinson’s disease, cognitive deficits secondary to multiple sclerosis, and age-related cognitive decline without dementia, all warrant systematic study. Additionally, pediatric applications require developmentally sensitive P300 paradigms and normative data spanning childhood and adolescence [5, 20, 83, 84, 125, 126, 127].

Finally, as P300 biomarkers begin to be implemented in clinical practice, consideration of equity and access issues will be crucial. Research studies utilizing diverse populations will be needed to ensure that the P300 biomarker is valid across demographic groups. Development of affordable, usable P300 technologies for low-resource environments will be important for expanding access to the P300 biomarker. Strategies for increasing access to P300 biomarkers for underserved populations will be important to avoid widening existing disparities in neuropsychiatric care [128, 129, 130, 131].

### 4.11 Limitations

This systematic review has several limitations that should be acknowledged. First, substantial methodological heterogeneity was observed across the 52 included studies, including variability in P300 paradigms (auditory oddball, visual oddball, Go/No-Go, monetary incentive delay), EEG acquisition parameters (2 to 64 channels, variable reference schemes), and population characteristics (age ranges from childhood to older adulthood, diverse diagnostic criteria, medication status, and comorbidities). This heterogeneity limits direct cross-study comparisons and contributes to statistical heterogeneity (I² = 67.3%). Second, risk of bias concerns were identified, with moderate to high performance bias in approximately 69% of studies due to challenges in blinding participants to neurostimulation or behavioral interventions. Sample sizes varied considerably, with some studies potentially underpowered for detecting reliable effects. Third, study design variability affected the interpretation of findings. Control conditions ranged from sham stimulation to waitlist controls, treatment duration varied from single sessions to 12 weeks, and follow-up periods ranged from immediately post-treatment to 6 months. Fourth, this review was restricted to English-language peer-reviewed articles published between January 2020 and August 2025, potentially excluding relevant studies published in other languages or timeframes. Finally, the absence of standardized normative data and established clinical cutoffs for P300 parameters limits the clinical translation of findings. Despite these limitations, the predominance of randomized controlled designs and objective EEG-based outcomes provides methodological strengths that increase confidence in the observed treatment effects.

## 5. Conclusions

This systematic review of 52 randomized and controlled studies establishes P300 event-related potentials as valid cognitive biomarkers with considerable potential for clinical evaluation and treatment monitoring across neurological and neuropsychiatric disorders. Evidence demonstrates that P300 parameters—latency reflecting cognitive processing speed and amplitude measuring attentional resource allocation—consistently correlate with cognitive impairment across six diagnostic categories: dementia spectrum disorders, acquired brain injury and disorders of consciousness, mood and anxiety disorders, neurodevelopmental and attention disorders, psychotic disorders and addiction, and chronic neurological conditions. These findings position P300 as a flexible transdiagnostic biomarker applicable to diagnosis, prognosis, treatment monitoring, and pharmacodynamic assessment.

P300 abnormalities, characterized by prolonged latency and reduced amplitude, consistently defined clinical populations across all research questions with moderate-to-large effect sizes. In dementia spectrum disorders (n = 11 studies), P300 latency variability differentiated mild cognitive impairment from healthy aging with sufficient sensitivity for population-level screening. In disorders of consciousness (n = 10 studies), P300 waveform presence served as an objective measure of residual cognitive function, complementing behavioral assessments. In mood and anxiety disorders (n = 7 studies), baseline amplitude demonstrated unique predictive value for treatment completion and future depression development. In psychotic disorders and addiction (n = 12 studies), P300 served as both a trait and a state marker, differentiating substance dependence subtypes and correlating with craving severity. These disorder-specific profiles demonstrate P300’s utility for both transdiagnostic assessment and differential characterization.

A critical finding was P300’s consistent responsiveness to therapeutic interventions across treatment modalities. Neuromodulation interventions (tDCS and rTMS) produced the most robust P300 normalization, with latency reductions and amplitude increases corresponding to improvements on standardized measures (MMSE, MoCA, CRS-R). Cognitive training interventions yielded reliable P300 improvements in populations with dementia and stroke rehabilitation. Pharmacotherapy effects were disorder-dependent, with positive outcomes for fosgonimeton in Alzheimer’s disease and methylphenidate in ADHD, but negative outcomes with certain antiepileptic medications. Neurofeedback results were variable, suggesting effects may depend on successful learning rather than the intervention modality itself.

Importantly, P300 changes may provide earlier indicators of treatment response than standard clinical evaluations. Latency reductions were observed within 3 days following acute metabolic intervention and within hours of single-dose pharmacologic administration, with broader normalization occurring within 2–6 weeks of treatment initiation. This temporal advantage over traditional assessments, which typically require 4–8 weeks, could enable early identification of non-responders and timely treatment modifications.

Several factors support near-term clinical translation. Validation of portable EEG systems, including two-channel configurations for mild cognitive impairment detection, demonstrates that meaningful P300 assessment does not require laboratory-grade equipment or specialized neurophysiology expertise. Strong correlations between P300 parameters and established clinical measures provide concurrent validity supporting P300 as an adjunct to existing assessment protocols. Applications with sufficient supporting evidence include consciousness assessment in disorders of consciousness, treatment response monitoring, and cognitive screening in dementia populations.

However, methodological variability across studies—including differences in acquisition parameters, paradigm specifications, and reporting practices—presents barriers to cross-study comparison and meta-analytic integration. Standardization in technical protocols, elicitation paradigms, and reporting guidelines is essential for enhancing reproducibility and clinical utility. Multi-site validation studies are necessary to demonstrate P300 biomarker reliability across diverse settings and populations.

Given the global burden of neuropsychiatric disorders—approximately 55 million people with dementia and 280 million with depression worldwide—P300 represents a potentially valuable tool for objective, scalable cognitive assessment extending beyond specialty care to primary care and community settings. Applications in early detection, such as identifying mild cognitive impairment before dementia onset or adolescents at risk for depression, could enable preventive interventions with significant public health benefit.

Future research priorities include: (1) large-scale, multi-center validation studies with age-specific reference values; (2) prospective evaluation of P300-guided treatment algorithms in real-world clinical environments; (3) development of user-friendly EEG devices with automated analysis pipelines; (4) mechanistic studies integrating P300 with other neuroimaging modalities; and (5) cost-effectiveness analyses to inform healthcare policy decisions.

P300 has the potential to contribute significantly to precision psychiatry by enabling treatment prediction, response monitoring, and clinical trajectory forecasting—replacing trial-and-error approaches with biomarker-based treatment selection. As computational methods evolve, P300-based algorithms will likely play increasing roles in clinical decision-making for neuropsychiatric disorders.

In conclusion, this systematic review demonstrates that P300 event-related potentials are valid, treatment-responsive cognitive biomarkers with established clinical utility across multiple neurological and neuropsychiatric conditions. The consistency of findings across diagnostic categories, correlations with established clinical measures, and responsiveness to diverse interventions support P300’s readiness for clinical translation in specific applications. Realizing P300’s full potential will require continued methodological standardization, multi-site validation, technological advancement, and implementation research. With coordinated interdisciplinary collaboration, P300 biomarkers can contribute to transforming mental health care toward greater objectivity, proactivity, and precision.

## Data Availability

The datasets used and analyzed during the current study are available from the corresponding author on reasonable request

## Abbreviations

AD, Alzheimer’s disease; ADHD, attention-deficit/hyperactivity disorder; ADs, antidepressants; AEDs, antiepileptic drugs; ASMs, antiseizure medications; ASD, autism spectrum disorder; ASSR, auditory steady-state response; ATA, atmospheres absolute; AUD, alcohol use disorder; AVLT-H, Auditory Verbal Learning Test–Huashan version; BCI, brain-computer interface; BDNF, brain-derived neurotrophic factor; BI, Barthel Index; BNT, Boston Naming Test; BRIEF2, Behavior Rating Inventory of Executive Function, Second Edition; BWL, blue-enriched white light; CACT, computer-assisted cognitive training; CBZ, carbamazepine; CDT, Clock Drawing Test; CI, confidence interval; CRS-R, Coma Recovery Scale-Revised; CT, conventional treatment; cTBS, continuous theta burst stimulation; DHI, Dizziness Handicap Inventory; DLPFC, dorsolateral prefrontal cortex; DMN, default mode network; DOC, disorders of consciousness; EEG, electroencephalography; EMG-BF, electromyography biofeedback; ERD, event-related desynchronization; ERP, event-related potential; FIM, Functional Independence Measure; fMRI, functional magnetic resonance imaging; fNIRS, functional near-infrared spectroscopy; FRN, feedback-related negativity; FSS, Fatigue Severity Scale; FTND, Fagerström Test for Nicotine Dependence; GRADE, Grading of Recommendations, Assessment, Development and Evaluations; HAMA-14, Hamilton Anxiety Rating Scale, 14-item version; HAMD, Hamilton Depression Rating Scale; HAMD-24, Hamilton Depression Rating Scale, 24-item version; HBOT, hyperbaric oxygen therapy; HCs, healthy controls; HE, hepatic encephalopathy; HGF/MET, hepatocyte growth factor/mesenchymal-epithelial transition factor; IGD, internet gaming disorder; ITR, information transfer rate; iTBS, intermittent theta burst stimulation; LCF, Levels of Cognitive Functioning (Rancho Los Amigos Scale); LEV, levetiracetam; LOLA, L-ornithine and L-aspartate; LPP, late positive potential; MBI, Modified Barthel Index; MCI, mild cognitive impairment; MCS, minimally conscious state; MDD, major depressive disorder; MEG, magnetoencephalography; MHE, minimal hepatic encephalopathy; MMN, mismatch negativity; MMSE, Mini-Mental State Examination; MoCA, Montreal Cognitive Assessment; MPH, methylphenidate; MS, multiple sclerosis; ND, nicotine dependence; NF, neurofeedback; NS, non-significant; OCD, obsessive-compulsive disorder; PANSS, Positive and Negative Syndrome Scale; PET, positron emission tomography; PI, prediction interval; PRISMA, Preferred Reporting Items for Systematic Reviews and Meta-Analyses; PROSPERO, International Prospective Register of Systematic Reviews; PSD, post-stroke depression; PSCI, post-stroke cognitive impairment; RCT, randomized controlled trial; REML, restricted maximum likelihood; RoB, risk of bias; rTMS, repetitive transcranial magnetic stimulation; SCP, slow cortical potential; SCZ, schizophrenia; SDMT, Symbol Digit Modalities Test; SMD, standardized mean difference; SPA, smartphone addiction; SPS, Suicide Probability Scale; tACS, transcranial alternating current stimulation; TBI, traumatic brain injury; tDCS, transcranial direct current stimulation; TMT-A, Trail Making Test Part A; TMT-B, Trail Making Test Part B; TPM, topiramate; tRNS, transcranial random noise stimulation; VaD, vascular dementia; VCI, vascular cognitive impairment; VR, virtual reality; VRT, vestibular rehabilitation therapy.

## Supplementary Material

Supplementary material associated with this article can be found, in the online version, at https://doi.org/10.31083/RN49664.
