# Supplementary material for: P300 Event-Related Potentials as Cognitive Biomarkers in Neurological and Neuropsychiatric Disorders: A Systematic Review
Source: Rev Neurol. 2026 May 26;81(5):49664. doi: 10.31083/RN49664 (PMC13221677; doi:10.31083/RN49664)
Supplement: Supplementary file 1 [file 1576-6578-81-5-49664-s1.zip › PRISMA_2020_checklist.docx]

| **Section and Topic** | **Item #** | **Checklist item** | **Location where item is reported** |
| --- | --- | --- | --- |
| **TITLE P300 Event-Related Potentials as Cognitive Biomarkers in Neurological and Neuropsychiatric Disorders: A Systematic Review** | | |  |
| Title | 1 | Identify the report as a systematic review. | Page 1 |
| **ABSTRACT** | | |  |
| Abstract | 2 | See the PRISMA 2020 for Abstracts checklist. | Page 1 |
| **INTRODUCTION** | | |  |
| Rationale | 3 | Describe the rationale for the review in the context of existing knowledge. | Pages 2–3 (Section 1. Introduction) |
| Objectives | 4 | Provide an explicit statement of the objective(s) or question(s) the review addresses. | Page 3 (Section 1. Introduction, Research Questions RQ1–RQ6) |
| **METHODS** | | |  |
| Eligibility criteria | 5 | Specify the inclusion and exclusion criteria for the review and how studies were grouped for the syntheses. | Pages 4–5 (Section 2.3. Eligibility Criteria, including 2.3.1 Inclusion and 2.3.2 Exclusion Criteria) |
| Information sources | 6 | Specify all databases, registers, websites, organisations, reference lists and other sources searched or consulted to identify studies. Specify the date when each source was last searched or consulted. | Pages 3–4 (Section 2.1. Analytical Search Process; PubMed, Scopus, Web of Science, PsycINFO; January 2020–August 2025, inclusive of a supplementary updated search covering April–August 2025) |
| Search strategy | 7 | Present the full search strategies for all databases, registers and websites, including any filters and limits used. | Page 4 (Section 2.2. Search Strategy) |
| Selection process | 8 | Specify the methods used to decide whether a study met the inclusion criteria of the review, including how many reviewers screened each record and each report retrieved, whether they worked independently, and if applicable, details of automation tools used in the process. | Pages 3–4 (Section 2.1. Analytical Search Process); Pages 5–7 (Section 2.4. Risk of Bias Assessment; two independent reviewers) |
| Data collection process | 9 | Specify the methods used to collect data from reports, including how many reviewers collected data from each report, whether they worked independently, any processes for obtaining or confirming data from study investigators, and if applicable, details of automation tools used in the process. | Page 7 (Section 2.5. Data Extraction and Synthesis) |
| Data items | 10a | List and define all outcomes for which data were sought. Specify whether all results that were compatible with each outcome domain in each study were sought (e.g. for all measures, time points, analyses), and if not, the methods used to decide which results to collect. | Page 7 (Section 2.5. Data Extraction and Synthesis; P300 amplitude, latency, electrode site; clinical outcomes) |
|  | 10b | List and define all other variables for which data were sought (e.g. participant and intervention characteristics, funding sources). Describe any assumptions made about any missing or unclear information. | Page 7 (Section 2.5. Data Extraction and Synthesis; bibliographical info, study design, sample features, P300 paradigm, intervention characteristics) |
| Study risk of bias assessment | 11 | Specify the methods used to assess risk of bias in the included studies, including details of the tool(s) used, how many reviewers assessed each study and whether they worked independently, and if applicable, details of automation tools used in the process. | Pages 5–7 (Section 2.4. Risk of Bias Assessment; modified Cochrane RoB 2.0; two independent reviewers) |
| Effect measures | 12 | Specify for each outcome the effect measure(s) (e.g. risk ratio, mean difference) used in the synthesis or presentation of results. | Page 19 (Section 2.6.2. Quantitative Synthesis; standardized mean difference [SMD] with 95% CIs) |
| Synthesis methods | 13a | Describe the processes used to decide which studies were eligible for each synthesis (e.g. tabulating the study intervention characteristics and comparing against the planned groups for each synthesis (item #5)). | Page 7 (Section 2.5. Data Extraction and Synthesis; grouped by six research questions) |
|  | 13b | Describe any methods required to prepare the data for presentation or synthesis, such as handling of missing summary statistics, or data conversions. | Page 7 (Section 2.5. Data Extraction and Synthesis) |
|  | 13c | Describe any methods used to tabulate or visually display results of individual studies and syntheses. | Page 7 (Section 2.5. Data Extraction and Synthesis); Tables 2–6 (Pages 8–18, 29, 31, 32, 39); Figures 1–6 (Pages 5, 7, 30, 31, 32, 33) |
|  | 13d | Describe any methods used to synthesize results and provide a rationale for the choice(s). If meta-analysis was performed, describe the model(s), method(s) to identify the presence and extent of statistical heterogeneity, and software package(s) used. | Page 19 (Sections 2.6.1 Synthesis Approach & 2.6.2 Quantitative Synthesis; random-effects meta-analysis using REML estimator; I², τ², Cochran’s Q) |
|  | 13e | Describe any methods used to explore possible causes of heterogeneity among study results (e.g. subgroup analysis, meta-regression). | Page 19 (Section 2.6.2 Subgroup analyses by intervention type and diagnostic category); Page 39 (Section 4.9. Limitations of Current Research and Methodological Heterogeneity) |
|  | 13f | Describe any sensitivity analyses conducted to assess robustness of the synthesized results. | Page 19 (Section 2.6.3. Sensitivity Analysis; leave-one-out analyses for quantitative syntheses) |
| Reporting bias assessment | 14 | Describe any methods used to assess risk of bias due to missing results in a synthesis (arising from reporting biases). | Page 19 (Section 2.6.4. Publication Bias Assessment; funnel plot asymmetry and Egger’s regression test); Pages 5–7 (Section 2.4. Risk of Bias Assessment, Reporting Bias domain) |
| Certainty assessment | 15 | Describe any methods used to assess certainty (or confidence) in the body of evidence for an outcome. | Page 19 (Section 2.6.5. Certainty of Evidence; GRADE-informed assessment); Pages 38–39 (Section 4.7. Certainty of Evidence Assessment, Table 6) |
| **RESULTS** | | |  |
| Study selection | 16a | Describe the results of the search and selection process, from the number of records identified in the search to the number of studies included in the review, ideally using a flow diagram. | Pages 3–4 (Section 2.1. Analytical Search Process); Page 5 (Figure 1. PRISMA Flow Diagram) |
|  | 16b | Cite studies that might appear to meet the inclusion criteria, but which were excluded, and explain why they were excluded. | Page 4 (Section 2.1: 198 duplicates, 23 non-English, 36 pre-2020, 65 irrelevant titles); Pages 4–5 (Section 2.3.2 Exclusion Criteria); Page 5 (Figure 1. PRISMA Flow Diagram) |
| Study characteristics | 17 | Cite each included study and present its characteristics. | Pages 8–18 (Table 2. Research articles of systematic analysis, n = 52) |
| Risk of bias in studies | 18 | Present assessments of risk of bias for each included study. | Page 7 (Figure 2. Risk of bias assessment across 52 studies) |
| Results of individual studies | 19 | For all outcomes, present, for each study: (a) summary statistics for each group (where appropriate) and (b) an effect estimate and its precision (e.g. confidence/credible interval), ideally using structured tables or plots. | Pages 8–18 (Table 2); Pages 19–32 (Section 3. Results) |
| Results of syntheses | 20a | For each synthesis, briefly summarise the characteristics and risk of bias among contributing studies. | Pages 19–32 (Section 3. Results, by RQ1–RQ6); Page 7 (Figure 2. Risk of bias assessment) |
|  | 20b | Present results of all statistical syntheses conducted. If meta-analysis was done, present for each the summary estimate and its precision (e.g. confidence/credible interval) and measures of statistical heterogeneity. If comparing groups, describe the direction of the effect. | Pages 29–32 (Section 3.7. Summary of P300 Findings Across Disorders; Tables 3–5); Page 31 (Figure 4. Forest plot of P300 latency changes, k = 18, SMD = –0.72 [95% CI: –0.89, –0.55]) |
|  | 20c | Present results of all investigations of possible causes of heterogeneity among study results. | Pages 29–31 (Section 3.7. Subgroup heterogeneity by intervention type); Page 39 (Section 4.9. Limitations of Current Research and Methodological Heterogeneity) |
|  | 20d | Present results of all sensitivity analyses conducted to assess the robustness of the synthesized results. | Not conducted |
| Reporting biases | 21 | Present assessments of risk of bias due to missing results (arising from reporting biases) for each synthesis assessed. | Page 7 (Figure 2. Risk of bias assessment; Reporting Bias: 67.3% low risk, 21.2% moderate, 5.8% high, 5.8% unclear) |
| Certainty of evidence | 22 | Present assessments of certainty (or confidence) in the body of evidence for each outcome assessed. | Pages 38–39 (Section 4.7. Certainty of Evidence Assessment; Table 6 GRADE-informed certainty ratings) |
| **DISCUSSION** | | |  |
| Discussion | 23a | Provide a general interpretation of the results in the context of other evidence. | Pages 33–38 (Section 4. Discussion; Sections 4.1–4.6) |
|  | 23b | Discuss any limitations of the evidence included in the review. | Pages 34–35 (Section 4.2. Technical and Methodological Considerations in P300 Biomarker Validation); Page 39 (Section 4.9. Limitations of Current Research and Methodological Heterogeneity) |
|  | 23c | Discuss any limitations of the review processes used. | Page 39 (Section 4.9. Limitations of Current Research and Methodological Heterogeneity) |
|  | 23d | Discuss implications of the results for practice, policy, and future research. | Pages 35–40 (Section 4.3. Clinical Translation; Section 4.4. Public Health Applications; Section 4.5. Methodological Imperatives and Technical Frontiers; Section 4.6. Integrative Analysis and Future Trajectories; Section 4.7. Certainty of Evidence Assessment; Section 4.8. Implications for Public Health; Section 4.10. Future Directions in P300 Biomarker Research) |
| **OTHER INFORMATION** | | |  |
| Registration and protocol | 24a | Provide registration information for the review, including register name and registration number, or state that the review was not registered. | Page 3 (Section 2.1. Analytical Search Process; review not prospectively registered) |
|  | 24b | Indicate where the review protocol can be accessed, or state that a protocol was not prepared. | Page 3 (Section 2.1. Analytical Search Process; protocol developed a priori, available from the corresponding author upon reasonable request) |
|  | 24c | Describe and explain any amendments to information provided at registration or in the protocol. | Not applicable |
| Support | 25 | Describe sources of financial or non-financial support for the review, and the role of the funders or sponsors in the review. | Page 42 (Funding: “This research received no external funding”) |
| Competing interests | 26 | Declare any competing interests of review authors. | Page 42 (Conflicts of Interest: “The authors declare no conflicts of interest”) |
| Availability of data, code and other materials | 27 | Report which of the following are publicly available and where they can be found: template data collection forms; data extracted from included studies; data used for all analyses; analytic code; any other materials used in the review. | Page 42 (Availability of Data and Materials) |

*From:*  Page MJ, McKenzie JE, Bossuyt PM, Boutron I, Hoffmann TC, Mulrow CD, et al. The PRISMA 2020 statement: an updated guideline for reporting systematic reviews. BMJ 2021;372:n71. doi: 10.1136/bmj.n71
